# Comparison of 15 dinoflagellate genomes reveals extensive sequence and structural divergence in family Symbiodiniaceae and genus *Symbiodinium*

**DOI:** 10.1186/s12915-021-00994-6

**Published:** 2021-04-13

**Authors:** Raúl A. González-Pech, Timothy G. Stephens, Yibi Chen, Amin R. Mohamed, Yuanyuan Cheng, Sarah Shah, Katherine E. Dougan, Michael D. A. Fortuin, Rémi Lagorce, David W. Burt, Debashish Bhattacharya, Mark A. Ragan, Cheong Xin Chan

**Affiliations:** 1 Institute for Molecular Bioscience, The University of Queensland, Brisbane, QLD 4072 Australia; 2 Present address: Department of Integrative Biology, University of South Florida, Tampa, FL 33620 USA; 3 Present address: Department of Biochemistry and Microbiology, Rutgers University, New Brunswick, NJ 08901 USA; 4 Australian Centre for Ecogenomics, The University of Queensland, Brisbane, QLD 4072 Australia; 5 School of Chemistry and Molecular Biosciences, The University of Queensland, Brisbane, QLD 4072 Australia; 6 Commonwealth Scientific and Industrial Research Organisation (CSIRO) Agriculture and Food, Queensland Bioscience Precinct, St Lucia, QLD 4072 Australia; 7 Present address: Institute for Molecular Bioscience, The University of Queensland, Brisbane, QLD 4072 Australia; 8 UQ Genomics Initiative, The University of Queensland, Brisbane, QLD 4072 Australia; 9 Present address: School of Life and Environmental Sciences, The University of Sydney, Sydney, NSW 2006 Australia; 10 École Polytechnique Universitaire de l’Université de Nice, Université Nice-Sophia-Antipolis, 06410 Nice, Provence-Alpes-Côte d’Azur France; 11 Department of Biochemistry and Microbiology, Rutgers University, New Brunswick, NJ 08901 USA

**Keywords:** Dinoflagellates, Symbiosis, Coral symbionts, Genome evolution

## Abstract

**Background:**

Dinoflagellates in the family Symbiodiniaceae are important photosynthetic symbionts in cnidarians (such as corals) and other coral reef organisms. Breakdown of the coral-dinoflagellate symbiosis due to environmental stress (i.e. coral bleaching) can lead to coral death and the potential collapse of reef ecosystems. However, evolution of Symbiodiniaceae genomes, and its implications for the coral, is little understood. Genome sequences of Symbiodiniaceae remain scarce due in part to their large genome sizes (1–5 Gbp) and idiosyncratic genome features.

**Results:**

Here, we present de novo genome assemblies of seven members of the genus *Symbiodinium*, of which two are free-living, one is an opportunistic symbiont, and the remainder are mutualistic symbionts. Integrating other available data, we compare 15 dinoflagellate genomes revealing high sequence and structural divergence. Divergence among some *Symbiodinium* isolates is comparable to that among distinct genera of Symbiodiniaceae. We also recovered hundreds of gene families specific to each lineage, many of which encode unknown functions. An in-depth comparison between the genomes of the symbiotic *Symbiodinium tridacnidorum* (isolated from a coral) and the free-living *Symbiodinium natans* reveals a greater prevalence of transposable elements, genetic duplication, structural rearrangements, and pseudogenisation in the symbiotic species.

**Conclusions:**

Our results underscore the potential impact of lifestyle on lineage-specific gene-function innovation, genome divergence, and the diversification of *Symbiodinium* and Symbiodiniaceae. The divergent features we report, and their putative causes, may also apply to other microbial eukaryotes that have undergone symbiotic phases in their evolutionary history.

**Supplementary Information:**

The online version contains supplementary material available at 10.1186/s12915-021-00994-6.

## Background

Dinoflagellates are a diverse group of unicellular microalgae that are ubiquitous in marine and freshwater environments. In coral reefs, dinoflagellates of the family Symbiodiniaceae are the predominant photosynthetic symbionts of cnidarians (e.g. corals, sea anemones and jellyfish), giant clams, sponges and other microorganisms including foraminiferans and ciliates [1]. Symbiodiniaceae can contribute more than 90% of their carbon fixed via photosynthesis, to meet the energetic needs of the coral host [2].

Coral reef ecosystems worldwide are under severe threat from warming oceans and increased human activities in coastal areas [3]. A modest episodic increase in ocean surface temperature in this environment can result in oxidative damage and the decoupling of carbon flow between the symbiont and the host. Specifically, breakdown of the coral-dinoflagellate symbiosis (i.e. coral bleaching) puts the coral host at risk of starvation, disease and eventual death [4–6]. The global coral bleaching event between 2014 and 2017 is the longest on record, and episodic mass bleaching events continue to occur [7, 8]. Conservation strategies are urgently needed to maintain and restore existing coral reefs. The design of such interventions requires a multi-pronged approach to understand the role of each biotic component to sustain a healthy, resilient symbiosis [9–11]. Genomic resources have proven useful to inform conservation efforts [12], but genome-scale data for the coral reef symbionts remain scarce. The dearth of genome data from Symbiodiniaceae is explained by the relatively large sizes (1–5 Gbp) [13, 14], and complex, atypical structure of dinoflagellate genomes and chromosomes [15, 16].

The genetic diversity of Symbiodiniaceae can be explained by natural selection acting on genomes involved in a broad spectrum of symbiotic associations that vary in host specificity, transmission mode and permanence *in hospite* [17, 18] as well as by stochastic forces that can lead to genetic drift [19]. Symbiosis, or the lack thereof, has been implicated in the genome evolution of Symbiodiniaceae [20]. Most symbiotic species are thought to be facultative to some extent, with the potential to shift between a free-living motile stage (mastigote) and a spherical symbiotic stage (coccoid). The genomes of facultative and recent intracellular bacterial symbionts are usually dynamic, characterised by extensive structural rearrangements, intensified activity of transposable elements (TEs) and increased gene duplication that leads to the accumulation of pseudogenes [21, 22]. Symbiotic Symbiodiniaceae, predominantly facultative, are expected to display similar genomic features in contrast to free-living taxa; the latter group includes species that have thus far been found only in environmental samples and, in laboratory experiments, fail to successfully infect potential hosts [23, 24]. Based on current taxonomic classification, Family Symbiodiniaceae contains the greatest number of described species within the phylogenetically distinct Order Suessiales [25, 26] that also includes other free-living taxa such as *Polarella* [27] and *Sphaerodinium* [28].

Here, we generated draft genome assemblies from seven members of the genus *Symbiodinium*: two free-living, one opportunistic and four symbiotic isolates that represent distinct lifestyles of Symbiodiniaceae. In combination with other available data, we systematically compared whole-genome sequences of 15 dinoflagellate taxa (of which 13 are Symbiodiniaceae) to assess the divergence and genetic diversity of Symbiodiniaceae relative to those within the single genus of *Symbiodinium*. We uncovered extensive genome sequence divergence within *Symbiodinium* that is comparable to that among different genera of Symbiodiniaceae and gene families that may contribute to niche adaptation. This genetic diversity likely translates into more-complex interactions than previously thought between coral (and other) hosts and Symbiodiniaceae symbionts.

## Results

### High genome divergence among Symbiodiniaceae taxa

**Fig. 1:**
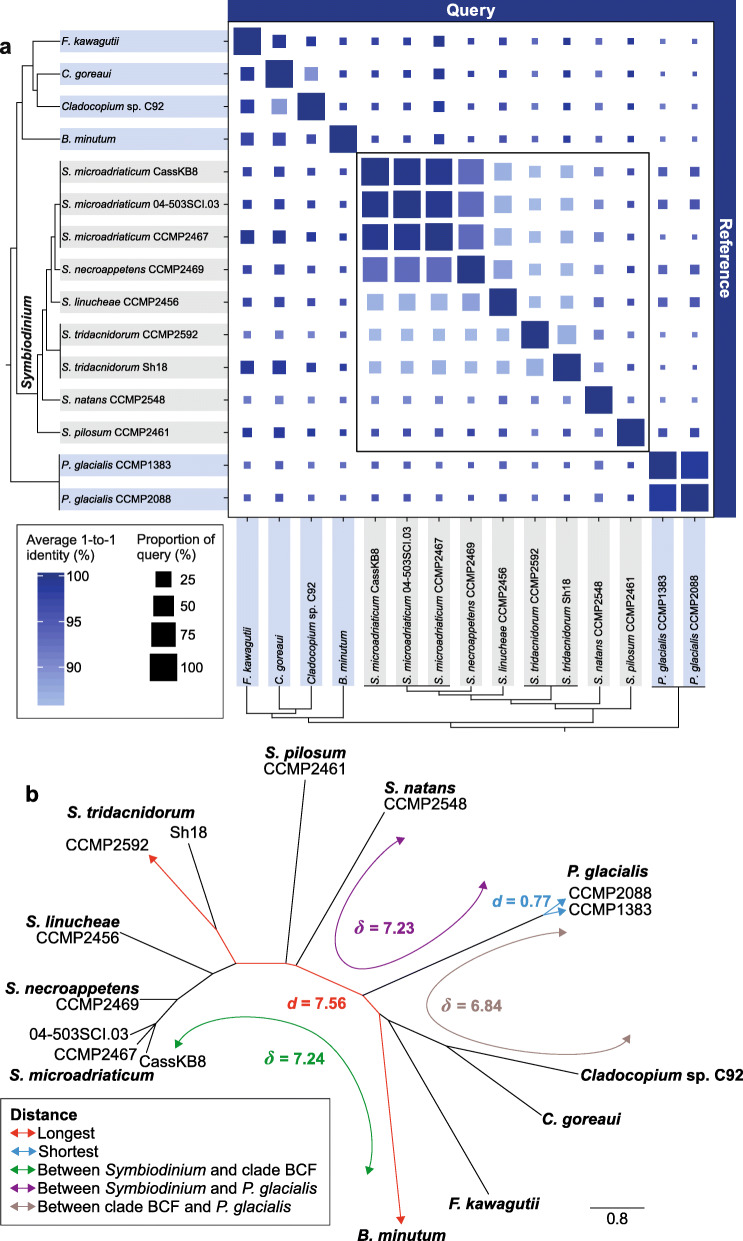
Genome divergence among Symbiodiniaceae. **a** Similarity between Symbiodiniaceae (and the outgroup *P. glacialis*) based on pairwise whole-genome sequence alignments. The colour of the square depicts the average percent identity of the best reciprocal one-to-one aligned regions (*I*) between each genome pair and the size of the square is proportional to the percent of the query genome that aligned to the reference (*Q*), as shown in the legend. The tree topologies on the left and bottom indicate the known phylogenetic relationship [26] among the isolates. Isolates in *Symbiodinium* are highlighted in grey, and their comparisons are highlighted in a bounded box. **b** Neighbour-joining tree based on 21-mers shared by genomes of Suessiales; branch lengths are proportional to the estimated distances. The shortest and longest distances (*d*) in the tree, as well as average distances (*δ*) among representative clades are shown following the bottom-left colour code. ‘Clade BCF’: clade including *B. minutum*, the two *Cladocopium* isolates, and *F. kawagutii*

We generated de novo genome assemblies for seven *Symbiodinium* isolates encompassing distinct lifestyles; two are hybrid assemblies incorporating both short- and long-read sequence data (Table 1 and Additional file 3: Supplementary Table 1). We included in our analysis available genome assemblies from six other Symbiodiniaceae (largely derived from short-read data) and two hybrid assemblies from the outgroup species *Polarella glacialis* (all in Order Suessiales; Additional file 3: Supplementary Table 2), totalling 15 dinoflagellate genomes. We assessed genome sequence similarity based on pairwise alignment of whole-genome sequences (see “Methods”). In each pairwise comparison, we assessed the overall percentage of the query genome sequence that aligned to the reference (*Q*), and the average percent identity of the reciprocal best one-to-one aligned sequences (*I*). Our results revealed extensive sequence divergence among these genomes at the order (Suessiales), family (Symbiodiniaceae) and genus (*Symbiodinium*) levels (Fig. 1a). As expected, genome pairs that exhibit the highest sequence similarity are isolates from the same species, e.g. between *S. microadriaticum* CassKB8 and 04-503SCI.03 (*Q* = 87.44%, *I* = 99.72%; CassKB8 as query), and between the two *P. glacialis* isolates (*Q* = 97.10%, *I* = 98.59%; CCMP1383 as query). In contrast, genome sequences of the two *S. tridacnidorum* isolates appear more divergent (*Q* = 30.07%, *I* = 87.18%; CCMP2592 as query). Remarkably, some genomes within *Symbiodinium* are as divergent as those of distinct genera: for instance, *Q* = 1.10% and *I* = 91.88% for *S. pilosum* compared against *S. natans* as reference, and *Q* = 1.03% and *I* = 92.15% for *S. tridacnidorum* CCMP2592 against *Cladocopium* sp. C92 (Fig. 1a).

**Table 1:** The seven *Symbiodinium* isolates for which de novo genome assemblies were generated in this study

	***S. microadriaticum*** CassKB8	***S. microadriaticum*** 04-503SCI.03	***S. tridacnidorum*** CCMP2592*	***S. linucheae*** CCMP2456	***S. necroappetens*** CCMP2469	***S. natans*** CCMP2548*	***S. pilosum*** CCMP2461
*ITS2* subtype	A1	A1	A3	A4	A13	–	A2
Lifestyle	Symbiotic	Symbiotic	Symbiotic	Symbiotic	Opportunistic	Free-living	Free-living
Host or source of origin	*Cassiopea* sp. (jellyfish)	*Orbicella faveolata* (stony coral)	*Heliofungia actiniformis* (stony coral)	*Plexaura homamalla* (octocoral)	*Condylactis gigantea* (anemone)	Open ocean	*Zoanthus sociatus* (zoanthid)
Collection site	Hawaii (Pacific)	Florida (Atlantic)	Coral Sea (Pacific)	Bermuda (Atlantic)	Jamaica (Caribbean)	Hawaii (Pacific)	Jamaica (Caribbean)
Overall G+C (%)	51.91	50.46	51.01	50.36	50.85	51.79	48.21
Number of scaffolds	67,937	57,558	6245	37,772	104,583	2855	48,302
Assembly length (bp)	813,744,491	775,008,844	1,103,301,044	694,902,460	767,953,253	761,619,964	1,089,424,773
N50 scaffold length (bp)	42,989	49,975	651,264	58,075	14,528	610,496	62,444
Max. scaffold length (Mbp)	0.38	1.08	4.01	0.46	1.34	3.40	1.34
Number of contigs	167,159	162,765	7913	141,380	157,685	4262	142,969
N50 contig length (bp)	10,400	11,136	356,695	11,147	11,420	358,021	17,506
Max. contig length (Mbp)	0.15	1.05	2.96	0.19	1.34	2.90	1.34
Gap (%)	1.15	1.44	0.02	1.35	0.56	0.02	0.79
Estimated genome size (bp)	1,120,150,369	1,052,668,212	1,287,259,774	914,781,885	1,007,022,374	740,100,732	1,993,912,458
Assembled fraction of genome (%)	72.65	73.62	85.71	75.96	76.26	100.03	54.64

An asterisk (*) denotes a hybrid genome assembly incorporating both short- and long-read sequence data. All other assemblies were generated using short-read sequence data

Scalable, alignment-free phylogenetic approaches [29], which bypass multiple sequence alignment and the computationally intensive tree-inference step, have been shown to yield accurate phylogenetic relationships among thousands of genome sequences from Bacteria and Archaea [30]. Adopting the approach by Bernard et al. [30], we assessed the proportion of shared *k*-mers (short, sub-sequences of defined length *k*) between each pair of genomes (optimised *k* = 21; see “Methods”) and calculated a pairwise distance (*d*) (Additional file 3: Supplementary Table 3). These distances were used to derive the phylogenetic relationship of the genomes as a neighbour-joining (NJ) tree (Fig. 1b) and as similarity networks at different similarity thresholds *T* (Additional file 2: Supplementary Figure 1). *Symbiodinium* isolates are about as distant from the other Symbiodiniaceae (mean$\delta$ = 7.24) as they are from the outgroup *P. glacialis* ($\delta$ = 7.23). The free-living *S. natans* [31] and *S. pilosum* are the most divergent from all others in the genus (*d* > 4.50 between either of them and one other *Symbiodinium* taxon; Additional file 3: Supplementary Table 3). The distance between *S. natans* and *S. pilosum* (*d* = 5.64) is similar to that between *F. kawagutii* and *C. goreaui* (*d* = 5.75) that are members of distinct genera. The genome sequence divergence among *Symbiodinium* isolates is further supported by the proportion of mapped sequence reads (Additional file 2: Supplementary Figure 2).

**Fig. 2:**
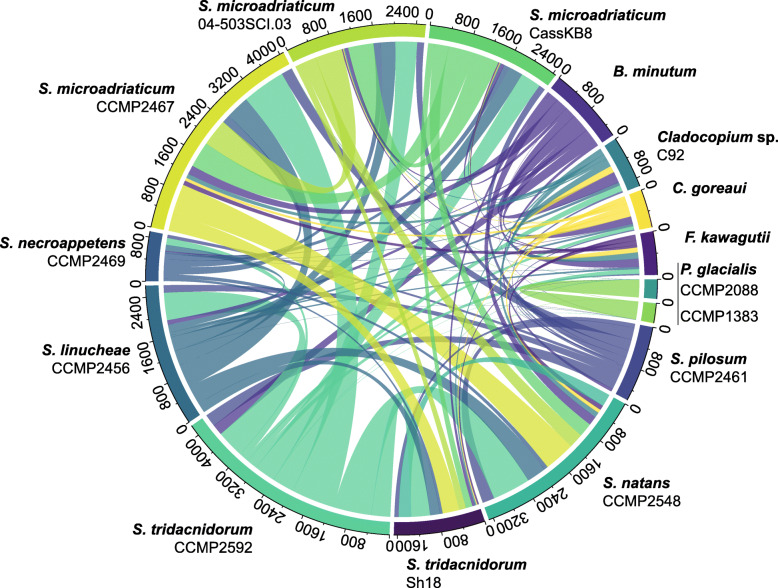
Conserved synteny of Suessiales genomes. Number of collinear syntenic gene blocks shared by pairs of Suessiales genomes. Gene blocks shared by more than two isolates are not shown

We used the same gene prediction workflow, customised for dinoflagellates [32, 33], for the seven *Symbiodinium* genome assemblies generated in this study and for the eight other assemblies included in our analyses (Additional file 3: Supplementary Table 4). To further assess genome divergence, we identified conserved synteny based on collinear syntenic gene blocks (see “Methods”). Figure 2 illustrates the gene blocks shared between all possible genome pairs. *S. microadriaticum* CCMP2467 and *S. tridacnidorum* CCMP2592 share the largest number of gene blocks (853 implicating 8589 genes). The 15 genome assemblies analysed here vary in sequence contiguity (Table 1, Additional file 3: Supplementary Table 2). However, the number of core conserved eukaryote genes identified in each assembly is comparable (mean 68.96% of BUSCO eukaryote_odb9 genes; Additional file 2: Supplementary Figure 3), suggesting similar data completeness among the genes that were predicted using a consistent workflow. Although we cannot dismiss the impact of assembly contiguity and completeness on our observations here (and results from the whole-genome alignment and *k*-mer analyses above), these results provide an overview of genome divergence at the level of species, genus and family.

**Fig. 3:**
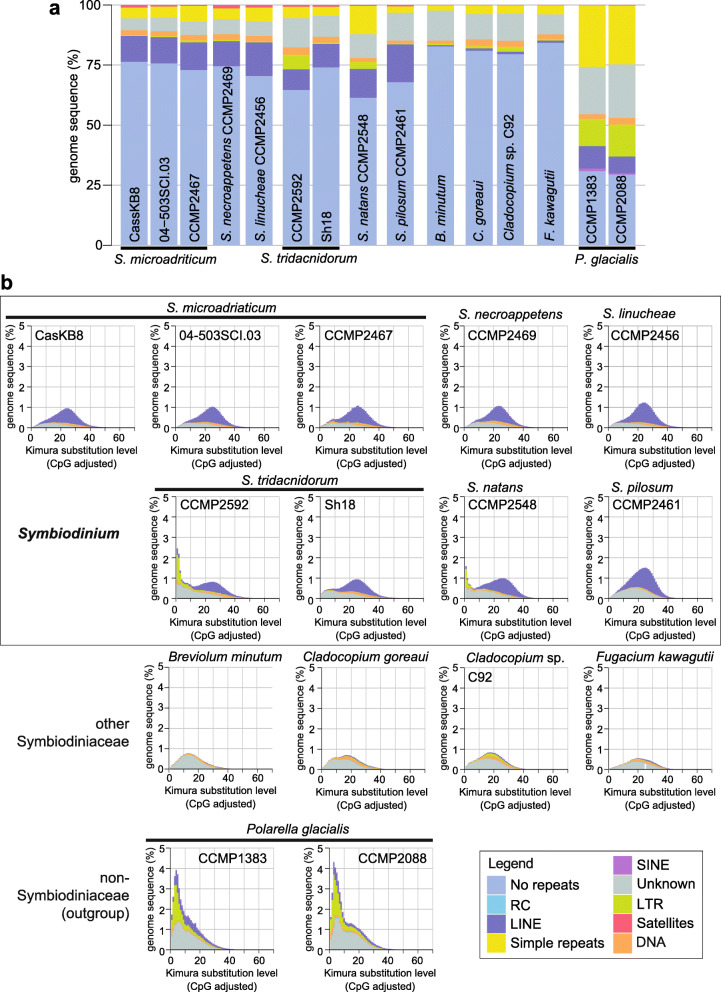
Repeat composition of Suessiales genomes. **a** Percentage of sequence regions comprising the major classes of repetitive elements, shown for each genome assembly analysed in this study. **b** Interspersed repeat landscape for each assembled genome. Both **a** and **b** follow the colour code shown in the legend. ‘No repeats’ refers to non-repetitive regions of the genome, ‘Unknown’ represents repeats that are not classified into any known types in the RepeatMasker database, including novel repeats

Figure 3a shows the composition of repeats for each of the 15 genomes. The repeat composition of the *P. glacialis* genome is distinct from Symbiodiniaceae, largely due to the prevalence of simple repeats [33]. To assess sequence divergence of repeats in each genome, Kimura distances [34], in which DNA transitions and transversions are assigned distinct substitution rates, were calculated for each repeat type (see “Methods”). Long interspersed nuclear elements (LINEs) in Symbiodiniaceae and in *P. glacialis* are highly divergent, with the Kimura distance centred between 15 and 40; these elements likely represent remnants of LINEs from an ancient expansion pre-dating the diversification of Suessiales [33, 35]. The proportion of these elements is substantially larger in the genomes of *Symbiodinium* and *P. glacialis* (the outgroup) than in those of other Symbiodiniaceae (Fig. 3b). For instance, LINEs comprise between 74.10 Mbp (*S. tridacnidorum* Sh18) and 96.90 Mbp (*S. linucheae*) of each of the *Symbiodinium* genomes, except in *S. pilosum*, where they encompass nearly twice as much in the genome (171.31 Mbp). In comparison, LINEs comprise, on average 7.49 Mbp of the genomes of the other Symbiodiniaceae (Additional file 2: Supplementary Figure 4). This implies that the remnants of LINEs were lost in the other Symbiodiniaceae genera. Interestingly, most LTRs and DNA transposons (recovered only in the hybrid assemblies incorporating both short- and long-read sequence data; Fig. 3b) are largely conserved (Kimura distance < 5), suggesting that they may remain active. The genome of *S. pilosum* has a nearly twofold increased abundance of LINEs, and a nearly twofold larger genome size estimate (1.99 Gbp) than other *Symbiodinium* genomes (Table 1 and Additional file 2: Supplementary Figure 4). Although this suggests whole-genome duplication or potential diploidy, we found no evidence to support either scenario (Additional file 2: Supplementary Figure 5). The prevalence of repetitive regions in *S. pilosum*, however, would explain in part why the total assembled bases of the genome constitute only 54.64% of the estimated genome size (Table 1). Our results reveal high sequence and structural divergence, and variable genome sizes among these *Symbiodinium* taxa, and compared to other Symbiodiniaceae, a distinct composition of repeats.

### Gene features of Symbiodiniaceae genomes

**Fig. 4:**
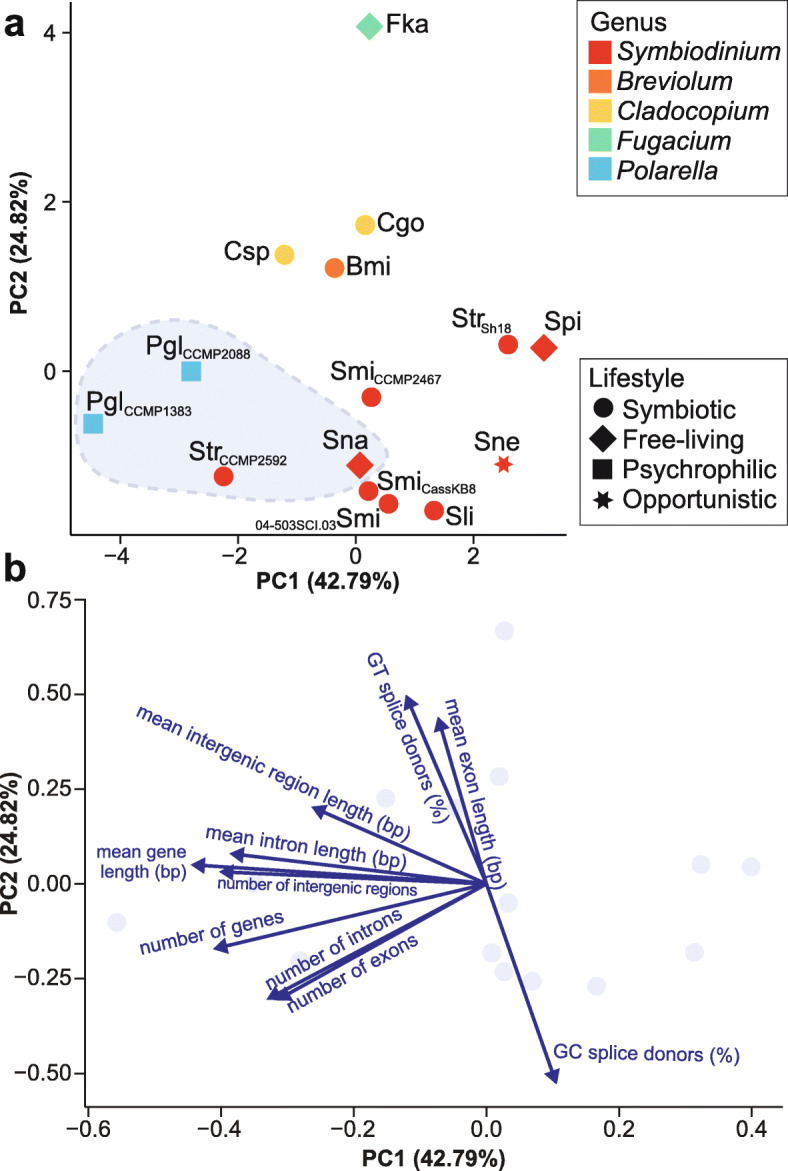
Gene features in Symbiodiniaceae genomes. **a** Principal component analysis (PCA) based on metrics of predicted genes from the analysed 15 genomes. Data points are coloured by genus and shaped by lifestyle according to the legends to the right. Data points enclosed in a light blue area correspond to isolates with hybrid genome assemblies. Smi: *S. microadriaticum*, Sne: *S. necroappetens*, Sli: *S. linucheae*, Str: *S. tridacnidorum*, Sna: *S. natans*, Spi: *S. pilosum*, Bmi: *B. minutum*, Cgo: *C. goreaui*, Csp: *Cladocopium* sp. C92, Fka: *F. kawagutii*, Pgl: *P. glacialis*. Isolate name is shown in subscript for those species with more than one isolate. **b** Loading plot showing the contribution of the distinct gene metrics employed for the PCA to PC1 and to PC2

Differences among predicted genes of Symbiodiniaceae have been attributed to phylogenetic relationships and to the implementation of distinct gene prediction approaches [32]. Our principal component analysis (PCA) approach, based on metrics of genes (Additional file 3: Supplementary Table 4) that were predicted using the same workflow (see “Methods”), revealed substantial variation within the genus *Symbiodinium* (Fig. 4a), and the contributions of the distinct metrics to this variation (Fig. 4b). We noted that the observed variation in gene architecture can be associated with one or more factors: (1) phylogenetic relationship, (2) the type of sequence data used for genome assembly and the consequent assembly quality and (3) lifestyle of the isolates. The variation resulting from the phylogenetic relationship among the genomes is illustrated by the separation of distinct genera along PC2 (explaining 24.82% of the variance). The metrics contributing the most to PC2 are associated with proportion of splice donors (Fig. 4b). The type of sequence data used for genome assembly and assembly quality is reflected along PC1 (explaining 42.79% of the variance). For instance, taxa for which hybrid assemblies were available (incorporating both short-read and long-read sequence data), i.e. the free-living *S. natans* and *P. glacialis*, and the symbiotic *S. tridacnidorum* CCMP2592, are distributed between −4.5 and 0.1 along PC1 (Fig. 4a). The distribution of the symbiotic *Symbiodinium* is limited (between 0.5 and 1.5 of PC1), with the exception of the two *S. tridacnidorum* isolates, for which the genome assemblies are of distinct quality, i.e. the high-quality hybrid assembly of CCMP2592 (Table 1) compared to the draft assembly of Sh18 that is fragmented (Additional file 3: Supplementary Table 2) and incomplete (Additional file 2: Supplementary Figure 3). In addition, the opportunistic *S. necroappetens* and free-living *S. pilosum* are distributed at > 2 along PC1 (Fig. 4a). These observations suggest that distinct lifestyles may contribute to differences in Symbiodiniaceae gene architecture.

The predicted coding sequences (CDS) among *Symbiodinium* taxa exhibit biases of nucleotide composition at the different codon positions (Additional file 2: Supplementary Figure 6) and in codon usage (Additional file 2: Supplementary Figure 7). The G+C content among CDS (Additional file 3: Supplementary Table 4) and among third codon positions (Additional file 2: Supplementary Figure 6) varies slightly but is generally higher relative to the overall G+C content (Table 1 and Additional file 3: Supplementary Table 2). This is consistent with the results previously reported for genomes and transcriptomes of Symbiodiniaceae [36]. Of all *Symbiodinium* isolates, *S. microadriaticum* CassKB8 and 04-503SCI.03 contain the largest number of CDS with a strong codon preference; *S. microadriaticum* CCMP2467 has the least (Additional file 2: Supplementary Figure 7). These observations highlight the genetic variation not only within a single genus, but also within a single species.

### Gene-family evolution

**Fig. 5:**
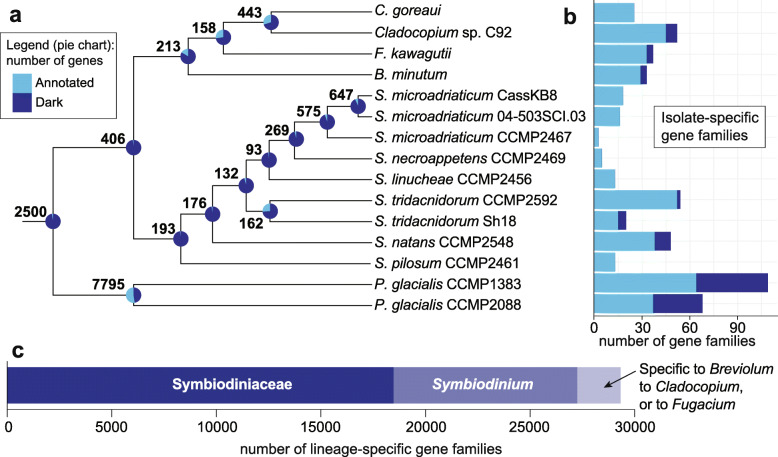
Number of gene families and conserved dark genes in Suessiales. **a** Tree topology shows the phylogenetic relationship of the 15 Suessiales isolates, follows the species tree inferred based on 28,116 gene families containing four or more sequences from any isolates, rooted with *P. glacialis* as outgroup to Symbiodiniaceae. At each node, the total number of families that include genes from all diverging isolates (and not others) is shown; the proportion of dark genes among all genes in these families are shown as a pie chart. **b** Number of isolate-specific gene families for each isolate, showing the proportion of families that are dark. **c** Number of gene families specific to Symbiodiniaceae, *Symbiodinium*, and other genera

Using all 555,682 predicted protein sequences from the 15 genomes, we inferred 42,539 homologous protein sets of size ≥ 2 (see “Methods”); here, we refer to these sets as gene families. Of these families, 2500 (5.88%) contain genes from all 15 Suessiales isolates, 406 are exclusive and common to all 13 Symbiodiniaceae isolates, and 193 are exclusive and common to all nine *Symbiodinium* isolates (Fig. 5a). Remarkably, predicted proteins coded by the vast majority of these conserved, lineage-specific genes (> 95% of almost all core genes at each internal node within *Symbiodinium* in Fig. 5a) do not share significant sequence similarity to any protein sequences in the UniProt database. We consider these as “dark” genes, i.e. they are either novel or too highly diverged to identify putative homologues in existing data; some families of dark genes appear to be specific to some isolates (Fig. 5b). Although proteins of *S. microadriaticum* CCMP2467 are available in UniProt (to which we expected many *Symbiodinium* proteins would share significant sequence similarity), the proportion (> 90%) of dark genes among the core genes of *Symbiodinium* remains higher than those among the core genes for other genera, e.g. the node encompassing *Breviolum*, *Cladocopium* and *Fugacium* (83.56% dark genes; Fig. 5a). This result suggests that the function of core genes in *Symbiodinium* species is largely unknown and that the divergence of gene sequences has been more extensive among the *Symbiodinium* isolates than among the other Symbiodiniaceae genera. Although these observations also reflect the dearth of dinoflagellate sequences in UniProt (and public databases in general), the presence of highly divergent homologues implies lineage-specific functional innovation, lending support to the observation of conserved, lineage-specific dark genes in dinoflagellates [37, 38]. Conserved dark genes were also reported in core genes of other eukaryote lineages including green algae [39] and the haptophyte *Emiliania* [40].

The species tree inferred using OrthoFinder (see “Methods”) based on the homologous protein families (Fig. 5a) is in complete topological congruence to the accepted Symbiodiniaceae phylogeny reconstructed using large subunit rRNA [26]. Our alignment-free tree (Fig. 1b) is also topologically congruent to the two phylogenies (in Fig. 5a and in [26]), except for the branching order of *S. natans* and *S. pilosum* basal to the *Symbiodinium* clade. This result demonstrates, in the case of Symbiodiniaceae and despite the extensive genome divergence, a strong consensus of phylogenetic signal exhibited by the different datasets (i.e. single marker gene, homologous protein families or whole-genome sequences), and the power of scalable alignment-free approaches for inferring phylogenetic relationships comprehensively using whole-genome sequences rather than a small subset of genes.

Of the 42,539 families, 18,453 (43.38%) contain genes inferred to be specific to Symbiodiniaceae at the permissive identity thresholds used. Interestingly, more (8828) gene families are specific to sequenced isolates of *Symbiodinium* than to sequenced isolates of all the other Symbiodiniaceae combined (2043 are specific to *Breviolum*, *Cladocopium* and *Fugacium* isolates; Fig. 5c). Although the simplest explanation is that substantially more gene families have been gained (or preserved) in *Symbiodinium* than in the other three genera, this may also be caused by the overrepresentation of this genus in our analysis. A previous study reported that substantially more gene families are specific to the clade encompassing *Breviolum*, *Cladocopium* and *Fugacium* (26,474) than are specific to *Symbiodinium* (3577) [36]. We consider our results here to be more reliable than those based largely on transcriptome data [36], in which gene numbers can be overestimated due to differential transcription, transcript isoforms, RNA editing and intrinsically shorter sequences. The smaller number of gene families shared among Symbiodiniaceae found here (i.e. 18,453 compared to 76,087 in González-Pech et al. [36]) reflects our more-conservative approach based on whole-genome sequence data, and our use of larger and/or higher-quality datasets. Nonetheless, our observations support the notion that the evolution of gene families has contributed to the diversification of Symbiodiniaceae [36].

### Gene functions related to symbiosis and stress response are less abundant in symbiotic Symbiodiniaceae

We examined the functions annotated for the predicted genes from all 15 Suessiales genomes based on the annotated Gene Ontology (GO) terms and protein domains. A recent study, focusing on the transcriptomic changes in *Cladocopium* sp. following establishment of symbiosis with coral larvae [41], compiled a list of symbiosis-related gene functions in Symbiodiniaceae. We searched for these functions and assessed their relative abundance in each analysed genome (see “Methods”) (Additional file 2: Supplementary Figure 8). GO terms related to N-glycan processing, immune response, transmembrane transport and the metabolism of carbohydrate, nitrogen and lipid were recovered at lower relative abundance in the prominent symbiotic lineages (i.e. *B. minutum*, *C. goreaui* and *Cladocopium* sp. C92) than in others. Genes encoding various Pfam domain types of ankyrin and tetratricopeptide repeat show varied relative abundances in the different lineages, supporting the distinct functions of these domains in specific host-symbiont recognition [41, 42].

Compared to the symbiotic *Breviolum* and *Cladocopium* lineages, we observed a higher relative abundance of gene functions related to DNA damage repair and cell division in genomes of *Symbiodinium*, *Fugacium* and *Polarella* (Additional File 2: Supplementary Figure 9). This is consistent with results from earlier studies [36, 43], lending support for sexual reproduction [35, 44, 45] and recombination as a contributing factor to the genetic diversity of Symbiodiniaceae [46–50]. Sexual reproduction has been described in other dinoflagellates [51]. A recent study [45] revealed that Symbiodiniaceae have the genetic capacity for sexual reproduction, potentially via an alternative mechanism for chromosomal crossover. In the genomes of *B. minimum* and the two *Cladocopium* isolates, we also observed a lower relative abundance of GO terms and protein domains associated with stress response, photosynthesis, motility, and phagocytosis (Additional file 2: Supplementary Figure 9), suggesting there are fewer genes encoding these functions in the symbiotic lineages compared to other lineages that include species with a non-symbiotic lifestyle (i.e. *Symbiodinium*, *F. kawagutii* and the outgroup *P. glacialis*), or an evolutionary trajectory of genome reduction that is expected in obligate intracellular symbionts [20, 22]. The prevalence of cold-shock DNA-binding and bacteriorhodopsin domains in *P. glacialis* is consistent with the finding of highly duplicated gene families encoding these functions in the genome [33] and highlights the adaptation of this species to extreme cold and low-light environments.

We assessed the potential impact of organellar genome sequences in the seven de novo assembled *Symbiodinium* genomes on our results (see “Methods”). For each genome, we found no more than 0.63% of assembled genome sequences that are putatively plastid or mitochondrial, and no gene models were predicted in these sequences (Additional file 3: Supplementary Table 5). Therefore, the contribution of organelle DNA to our results is negligible.

### Can symbiosis drive genome evolution in Symbiodiniaceae?

To inspect genome divergence potentially associated with a symbiotic lifestyle, we compared the two high-quality assemblies of (a) the symbiotic *S. tridacnidorum* [52] CCMP2592 that was isolated from a stony coral from the Great Barrier Reef and (b) the free-living *S. natans* [31] CCMP2548 (synonym HA3–5) isolated from sediment at the beach of Coconut Island, Hawaii [53]. Both of these genomes were generated using a combination of short- and long-read sequence data (Table 1 and Additional file 3: Supplementary Table 1). *S. tridacnidorum* encompasses isolates in *ITS2*-type A3 that are predominantly symbionts of giant clams in the Indo-Pacific [26]. Although the nature of this symbiosis is extracellular, *S. tridacnidorum* can also establish intracellular symbiosis with cnidarian hosts, both in experimental settings and in nature [52]. On the other hand, *S. natans* (the type species of the genus) is free-living. *S. natans* occurs frequently in environmental samples, exhibits a widespread distribution and, thus far, has not been shown to colonise cnidarian hosts [26, 31].

**Fig. 6:**
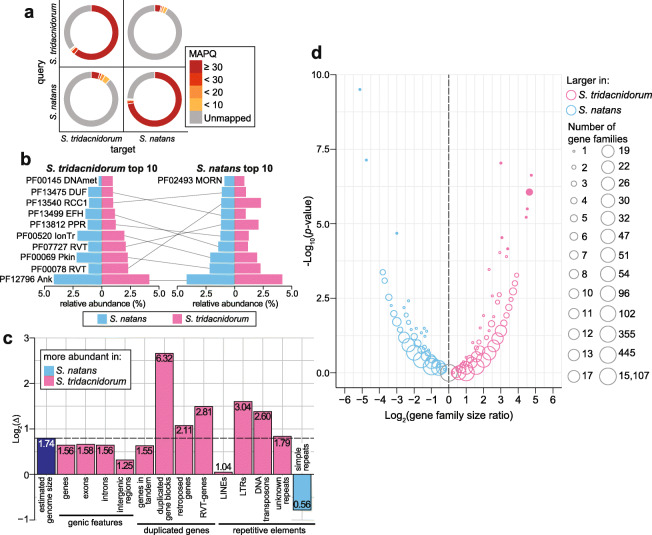
Comparison of *S. tridacnidorum* and *S. natans* genomes. **a** Mapping rate of filtered read pairs generated for each species against the assembled genomes of itself and of the counterpart: *S. tridacnidorum* (St) versus *S. natans* (Sn). **b** Top ten most abundant protein domains recovered, sorted in decreasing relative abundance (from bottom to top) among proteins of St (left) and those of Sn (right). The abundance for each domain in both genomes is shown in each chart for comparison. Domains common among the top ten most abundant for both species are connected with a line between the charts. ‘MORN’: MORN repeat, ‘RCC1’: Regulator of chromosome condensation repeat, ‘RVT’: reverse transcriptase, ‘DUF’: domain of unknown function, ‘PPR’: pentatricopeptide repeat, ‘EFH’: EF-hand, ‘IonTr’: ion transporter, ‘Pkin’: protein kinase, ‘Ank’: ankyrin repeat, ‘DNAmet’: C-5 cytosine-specific DNA methylase. **c** Contribution of genomic features to the distinct composition of *S. tridacnidorum* and *S. natans* genomes, based on the ratio (Δ) of the total length of the implicated sequence region in *S. tridacnidorum* to the equivalent length in *S. natans*, shown in log_2_ scale. The ratio of the estimated genome sizes is shown as reference (marked with a dashed line). The untransformed Δ for each feature is shown in its corresponding bar. A genome feature with Δ greater than the reference likely contributed to the discrepancy of genome sizes. Bars are coloured based on the genome in which they are more abundant as shown in the legend. Pseudogenes are not included in this plot. **d** Volcano plot comparing gene-family sizes against Fisher’s exact test significance (*p* value). The colour of the circles indicates the species in which those gene families are larger according to the top-right legend; families recovered only in one genome but not in the other are not shown. The number of gene families with the same ratio and significance is represented with the circle size following the bottom-right legend. Filled circles represent size differences that are considered statistically significant (adjusted *p* ≤ 0.05)

Compared to other genome assemblies generated using only short-read data (Table 1), these two genome assemblies provide better-resolved repetitive genomic regions, allowing for a more-in-depth comparative analysis of these regions. The estimated genome size is 1.29 Gbp for *S. tridacnidorum* and 0.74 Gbp for *S. natans* (Table 1 and Additional file 3: Supplementary Table 6); the latter is the smallest reported for any symbiodiniacean genome to date, although we note that accuracy for *k*-mer-based genome size estimation is sensitive to *k* length and the abundance of repeats in the data. The two genomes are highly dissimilar; we observed a low read-mapping rate (< 15%) of read pairs from one genome dataset against the genome assembly of the counterpart, and vice versa (Fig. 6a). Only 14.70 Mbp (1.33%) of the genome sequence of *S. tridacnidorum* aligned to 11.84 Mbp (1.55%) of *S. natans* at 90% identity or greater. Figure 6b shows the most-represented protein domains in the two genomes; gene functions related to methylation and retrotransposition, previously reported in other Symbiodiniaceae [54], are more abundant in *S. tridacnidorum*, whereas functions related to ion transport are more prominent in *S. natans* (Additional file 1: Supplementary Note [55–57] and Additional file 3: Supplementary Table 7).

We assessed the genome features distinct to each species that may have contributed to the discrepancy in genome size. Specifically, we assessed, for each feature, the ratio (Δ) of the total length of the implicated sequence regions in *S. tridacnidorum* to the equivalent length in *S. natans* (Fig. 6c). The genome size estimate for *S. tridacnidorum* is 1.74 times larger than that for *S. natans* (Additional file 3: Supplementary Table 6); we use this ratio as a reference for comparison. Most of the examined genome features span a larger region in the genome of *S. tridacnidorum*, as expected. The Δ for each inspected genic feature (even for exons and introns separately) approximates 1.74. However, six features related to duplicated genes and repetitive elements have Δ > 1.74. The abundance of repeats characteristic of TEs (such as LINEs and LTRs; Fig. 6c) suggests the enhanced activity of retrotransposition in *S. tridacnidorum*. In both genomes, we recovered genes that encode relicts of dinoflagellate spliced leader (DinoSL) sequences indicating retroposition of genetic elements. These genes encode various enzymatic functions (Additional file 2: Supplementary Figure 10); the presence of DinoSL relicts suggests the importance of high transcription activity of these genes in genome evolution. We recovered putative DinoSL sequences in both genomes (496 on 350 scaffolds of *S. tridacnidorum*, and 244 on 211 scaffolds of *S. natans*; see “Methods”), some in DinoSL or DinoSL-5S rRNA tandem arrays, as previously described in dinoflagellates [58]. We also recovered genes that encode the reverse transcriptase domains indicating retrotransposition (Additional file 3: Supplementary Tables 8 and 9). Thus, gene duplication and repeats likely expanded in *S. tridacnidorum*, and/or contracted in *S. natans*, contributing to the genome size discrepancy (Additional file 2: Supplementary Figure 11).

Tandem duplication of exons and genes is common in dinoflagellates [33, 59], and may serve as an adaptive mechanism to enhance functions relevant for their biology. Whereas in some dinoflagellates, genes in tandem arrays contain hundreds of copies, e.g. up to 5000 copies of the peridinin-chlorophyll a-binding protein (PCP) gene in *Lingulodinium polyedra* [60], these arrays are not as prominent in the genomes of *S. tridacnidorum* and *S. natans* (Additional file 2: Supplementary Figure 12), with the largest array comprising 10 and 13 gene copies, respectively. The length of duplicated gene blocks is markedly longer in *S. tridacnidorum* than in *S. natans* (Δ = 6.32; Fig. 6c). This observation, and the number of gene-block duplicates in each of the two species, suggests that segmental duplication has occurred more frequently (or alternatively, has been more preserved) in the course of genome evolution of *S. tridacnidorum*. We found 23 syntenic collinear blocks within the *S. tridacnidorum* genome (i.e. within-genome duplicated gene blocks) implicating 242 genes in total. Of these genes, 20 encode protein kinase functions (Additional file 3: Supplementary Table 10) that are associated with distinct signalling pathways. In comparison, only five syntenic collinear blocks implicating 62 genes were found in the *S. natans* genome; these genes largely encode functions of cation transmembrane transport, relevant for the maintenance of pH homeostasis. Ankyrin and pentatricopeptide repeats are common in the predicted proteins of duplicated genes in both genomes.

Using all predicted genes, we inferred 58,541 homologous gene families, of which only 16,663 (28.46%) contain genes from both genomes. Duplicated genes can experience distinct fates [61]. If the function remains the same or changes slightly (e.g. through subfunctionalisation), the duplicated gene sequences will remain similar, resulting in gene-family expansion. We assessed the difference in gene-family sizes between *S. tridacnidorum* and *S. natans* using Fisher’s exact test (see “Methods”) and considered those with an adjusted *p* ≤ 0.05 to be significantly different (Fig. 6d). We observe no strong evidence for gene-family expansion in *S. tridacnidorum* compared to *S. natans*; only 20 families are significantly larger, including OG0000004 that putatively encodes protein kinases and glycosyltransferases that are necessary for the biosynthesis of glycoproteins, and OG0000013 that encodes ankyrin and transport proteins (Additional file 3: Supplementary Table 11). These functions are important for the recognition of and interaction with the host among symbiodiniacean symbionts [41, 42]. In comparison, five gene families were significantly larger in *S. natans* than in *S. tridacnidorum*, of which one (OG0000003) encodes a sodium-transporter and another (OG0000034) a transmembrane protein. Many genes in the expanded families encode retrotransposition functions in both genomes, lending support to the contributing role of retrotransposons in gene-family expansion in Symbiodiniaceae [54] and more broadly in dinoflagellates [38].

If novel beneficial functions of the gene copies emerge (i.e. neofunctionalisation), the sequence divergence between gene copies may become too large to be recognised as the same family. This scenario could at least partially explain the higher number of single-copy genes exclusive to *S. tridacnidorum* (25,649, of which only 13,189 [51.42%] are supported by transcriptome evidence) than those exclusive to *S. natans* (16,137, of which 13,320 [82.54%] supported by transcriptome evidence). Although most dinoflagellate genes are known to be constitutively expressed regardless of exposure to abiotic stress [62], it remains unclear if the genes with no transcriptome support are functional genes, or not expressed in the conditions used for culture. However, the annotated functions for single-copy genes exclusive to each genome are similar in both species (Additional file 3: Supplementary Table 12), suggesting the presence of highly diverged homologues. In contrast, duplicated genes can also undergo loss of function (i.e. nonfunctionalisation or pseudogenisation). Pseudogene screening in both genomes (see “Methods”) identified 183,516 putative pseudogenes in *S. tridacnidorum* and 48,427 in *S. natans*. The nearly four-fold difference in the number of pseudogenes between the two genomes further supports the notion that duplication events [61] are more frequent in *S. tridacnidorum* and may explain the lower proportion of genes with transcript support in this species (Additional file 3: Supplementary Table 4).

These results suggest that the extensive genomic divergence between the symbiotic *S. tridacnidorum* and the free-living *S. natans*, including the discrepancy in genome sizes, is attributed to TEs, genetic duplication, structural rearrangements and pseudogenisation. These genome features are common in facultative and recent intracellular bacterial symbionts and parasites [21, 22], and expected in symbiotic Symbiodiniaceae [20]. The abundance of pseudogenes and larger genome size of the symbiotic species suggest the lack of the evolutionary pressure to purge the excessive genetic content during prolonged symbiotic associations. This observation contrasts with the smaller genomes of parasitic dinoflagellates, e.g. the genome of *Amoebophrya ceratii* (87.7 Mbp) that encodes fewer (19,925) genes [63]. Additional high-quality genome data from free-living and symbiotic taxa are required to gain a clearer understanding of the evolutionary transition(s) between free-living and symbiotic lifestyles in Symbiodiniaceae, and the impacts of symbiosis on genome evolution in these taxa (see Additional file 1: Supplementary Note).

## Discussion

Our results reveal high sequence and structural divergence among genomes of *Symbiodinium*, and more broadly of the family Symbiodiniaceae. Genomic divergence in microbial eukaryotes has been associated with adaptation to specialised or harsh environments [33, 64], lifestyle specialisation [63] and the capacity to inhabit diverse environments [40]. Although sexual recombination likely contributes to the extensive genetic diversity of the family Symbiodiniaceae [45, 46, 50, 65], its limitation to highly similar (likely homologous) sequence regions renders unlikely its role as the sole driver of genome divergence. The evolutionary transition from a free-living to a symbiotic lifestyle can contribute to the loss of conserved synteny as consequence of large- and small-scale structural rearrangements [20]. The enhanced activity of mobile elements in the early stages of this transition can further disrupt synteny, impact gene structure and accelerate mutation rate [66, 67]. However, *S. natans* and *S. pilosum*, for which the free-living lifestyle appears to be ancestral, are still quite diverged from each other (Additional file 2: Supplementary Figure 1). Ancient events, such as changes of sea level or climate, are thought to have influenced diversification of Symbiodiniaceae [26, 68, 69] and may help explain the divergence of the extant lineages. These events may have severely reduced effective population size, thereby weakening selection, and allowing genetic drift to impact genome structure. For example, in a hypothetical scenario, a drastic drop of the sea level could have split the ancestral Symbiodiniaceae population into multiple sub-populations with very small population sizes. This could have enabled rapid genome divergence among the sub-populations that, in turn, evolved and diversified independently into the extant taxa. However, the capacity of dinoflagellates to form cysts under stress [70] could have counteracted this effect by facilitating dispersal. Dinoflagellates are estimated to have diversified ~190 million years ago (MYA) [71]. The diversification time of Symbiodiniaceae, recently revised at ~160 MYA [26], corresponds to the adaptive radiation of reef-building (scleractinian) corals ~240 MYA [72] and supports the notion that symbiosis impacted genome evolution of Symbiodiniaceae. Nevertheless, other evolutionary processes (e.g. natural selection, gene loss and/or lateral genetic transfer) contributing to genomic divergence in these taxa remain to be systematically investigated.

The evolutionary mechanisms underscoring Symbiodiniaceae diversity have been examined in earlier studies based on host specificity, coevolution, biogeography, ecology and marker-based phylogenies [18, 26, 50, 73–75]. Our work, based on whole-genome sequence data, emphasises how much genomic diversity can lie hidden beneath a “simple” morphology that may differ only subtly, for example, with respect to plate arrangements on the cell surface of Symbiodiniaceae [26]. Beyond neutral evolution, symbioses with corals and other hosts may be driving both morphological uniformity and massive genome divergence in this family. Understanding the evolutionary mechanisms that facilitate genome rearrangements and gene-function innovation in dinoflagellate symbionts remains an important step in evolutionary studies of the coral holobionts. The astonishing number of dark genes in *Symbiodinium* species signifies that, with available protein annotations, we are addressing only the tip of the iceberg of divergent and/or novel gene functions in these dinoflagellates. To reconstruct genomic changes and novel gene origins more robustly, the divergence of whole-genome sequences among *Symbiodinium* should be considered in the light of known and yet-to-be discovered biodiversity of this genus.

## Conclusions

Our results reveal that, among these dinoflagellates, the genome architecture that underlies symbiotic associations with corals and other organisms is very diverse. Therefore, a one-size-fits-all approach with Symbiodiniaceae for engineering environmentally robust corals may prove ineffective. A combination of in situ and culture-based studies are needed to address these and other outstanding questions about the evolution of dinoflagellate symbionts of coral reefs, both with respect to genome architecture and innovation of novel gene functions, and about the resilience of coral-dinoflagellate symbioses in changing environments. The wealth of data and insights we have generated elucidate how symbiosis may underpin molecular mechanisms that drive genome evolution and divergence of Symbiodiniaceae. In the future, these insights could be used to reconstruct lifestyle impact on genome evolution of other microbial eukaryotes that presently exist as obligate or facultative symbionts or may once have been in their evolutionary history.

## Methods

### *Symbiodinium* cultures

Single-cell monoclonal cultures of *S. microadriaticum* CassKB8 and *S. microadriaticum* 04-503SCI.03 were acquired from Mary Alice Coffroth (Buffalo University, New York, USA), and those of *S. natans* CCMP2548, *S. tridacnidorum* CCMP2592, *S. necroappetens* CCMP2469, *S. linucheae* CCMP2456 and *S. pilosum* CCMP2461 were purchased from the National Center for Marine Algae and Microbiota at the Bigelow Laboratory for Ocean Sciences, Maine, USA (Table 1). The cultures were maintained in multiple 100-mL batches (in 250-mL Erlenmeyer flasks) in f/2 (without silica) medium (0.2 mm filter-sterilised) under a 14:10 h light-dark cycle (90 μE/m^2^/s) at 25 °C. The medium was supplemented with antibiotics (ampicillin [10 mg/mL], kanamycin [5 mg/mL] and streptomycin [10 mg/mL]) to reduce bacterial growth.

### Nucleic acid extraction

Genomic DNA was extracted following the 2×CTAB protocol with modifications. *Symbiodinium* cells were first harvested during exponential growth phase (before reaching 106 cells/mL) by centrifugation (3000*g*, 15 min, room temperature (RT)). Upon removal of residual medium, the cells were snap-frozen in liquid nitrogen prior to DNA extraction, or stored at −80 °C. For DNA extraction, the cells were suspended in a lysis extraction buffer (400 μL; 100 mM Tris-Cl pH 8, 20 mM EDTA pH 8, 1.4 M NaCl), before silica beads were added. In a freeze-thaw cycle, the mixture was vortexed at high speed (2 min), and immediately snap-frozen in liquid nitrogen; the cycle was repeated 5 times. The final volume of the mixture was made up to 2% w/v CTAB (from 10% w/v CTAB stock; kept at 37 °C). The mixture was treated with RNase A (Invitrogen; final concentration 20 μg/mL) at 37 °C (30 min) and Proteinase K (final concentration 120 μg/mL) at 65 °C (2 h). The lysate was then subjected to standard extractions using equal volumes of phenol:chloroform:isoamyl alcohol (25:24:1 v/v; centrifugation at 14,000*g*, 5 min, RT) and chloroform:isoamyl alcohol (24:1 v/v; centrifugation at 14,000*g*, 5 min, RT). DNA was precipitated using pre-chilled isopropanol (gentle inversions of the tube, centrifugation at 18,000 *g*, 15 min, 4 °C). The resulting pellet was washed with pre-chilled ethanol (70% v/v), before stored in Tris-HCl (100 mM, pH 8) buffer. DNA concentration was determined with NanoDrop (Thermo Scientific), and DNA with A_230:260:280_ ≈ 1.0:2.0:1.0 was considered appropriate for sequencing. Total RNA was isolated from *Symbiodinium* cells in culture (4 weeks post-inoculum, at exponential growth) using the RNeasy Plant Mini Kit (Qiagen) following directions of the manufacturer. RNA quality and concentration were determined using Agilent 2100 BioAnalyzer.

### Genome sequence data generation and de novo assembly

All genome sequence data generated for all seven *Symbiodinium* isolates are detailed in Additional file 3: Supplementary Table 1. Short-read sequence data (2 × 150 bp reads, insert length 350 bp) were generated using paired-end libraries on the Illumina HiSeq 2500 and 4000 platforms at the Australian Genome Research Facility (Melbourne) and the Translational Research Institute Australia (Brisbane). Some of the paired-end libraries (insert length 250 bp) were designed such that the read pairs of 2 × 150 bp would overlap. Quality assessment of the raw paired-end data was done with FastQC v0.11.5, and subsequent processing with Trimmomatic v0.36 [76]. To ensure high-quality read data for downstream analyses, the paired-end mode of Trimmomatic was run with the settings: ILLUMINACLIP:[AdapterFile]:2:30:10 LEADING:30 TRAILING:30 SLIDING WINDOW:4:25 MINLEN:100 AVGQUAL:30; CROP and HEADCROP were run (prior to LEADING and TRAILING) when required to remove read ends with nucleotide biases. Overlapping read pairs (from the library with insert size of 250 bp) were merged with FLASH v1.2.11 [77]. Library adapters from the mate pair data were removed with NxTrim v0.41 [78]. De novo genome assembly was performed for all isolates with CLC Genomics Workbench v7.5.1 (https://digitalinsights.qiagen.com/) at default parameters and using the filtered read pairs and single-end reads. These genome assemblies were further scaffolded with transcriptome data (where applicable; see below) using L_RNA_scaffolder [79].

Long-read sequence data for *S. natans* and *S. tridacnidorum* were generated on a PacBio Sequel system at the Ramaciotti Centre for Genomics (Sydney). In combination, these data and the paired-end libraries (adding up to a coverage of 152-fold for *S. natans* and 200-fold for *S. tridacnidorum*) were incorporated in hybrid de novo genome assembly with MaSuRCA 3.3.0 [80], following the procedure described in the manual. PacBio subreads were filtered to a minimum length of 5 kb, and all other sequence data were used as input without being pre-processed, as recommended [80]. The genome assemblies were further scaffolded with transcriptome data generated in this study (see below) using L_RNA_scaffolder [79]. These hybrid genome assemblies are more contiguous than the draft assemblies generated using only short-read sequence data (above), thus are of higher quality. Short sequences (< 1000 bp) were removed from all assemblies.

### Estimation of genome size and ploidy

Genome size and sequence read coverage were estimated based on the *k*-mer frequency in short-read data, following earlier studies [33, 35]. Briefly, for each genome, *k*-mers were enumerated from the Illumina paired-end reads using Jellyfish v2.2.6 [81], independently for *k* = 17, 19, 21, 23, 25, 27, 29 and 31. For each *k*, genome size is estimated by dividing the total number of observed *k*-mers by the maximum fold-coverage of the *k*-mers as determined from the frequency distribution peak. The final genome size estimate is the mean size estimate derived from the different *k* values. Ploidy of a genome can also be estimated based on the frequency distribution of *k*-mers of short-read data [82]; a single peak suggests that the genome is likely haploid (single copy, *n*). In the scenario of a diploid (2*n*) genome or recent whole-genome duplication, two peaks would be observed, such that *k*-mer coverage of the second peak is exactly (or approximately) twofold compared to that of the first.

### Removal of putative microbial contaminants

To identify putative sequences from bacteria, archaea and viruses in the genome scaffolds, we followed the two-phase approach of Chen et al. [32]. In brief, we first searched the scaffolds (BLASTn) against a database of bacterial, archaeal and viral genomes from RefSeq (release 88); hits with *E* ≤ 10^−20^ and alignment bit score ≥ 1000 were considered as significant. We then calculated the proportion of bases in each scaffold covered by significant hits. Next, we assessed the added length of implicated genome scaffolds across different thresholds of these proportions, and the corresponding gene models in these scaffolds as predicted from available transcripts using PASA v2.3.3 [83] (see below), with a modified script available at https://github.com/chancx/dinoflag-alt-splice that recognises an additional donor splice site (GA), and TransDecoder v5.2.0 [83]. Any scaffolds with significant bacterial, archaeal or viral hits covering ≥ 5% of its length was considered as a putative contaminant and removed from the assembly. Additionally, the length of the remaining scaffolds was plotted against their G+C content; scaffolds (> 100 kb) with irregular G+C content (in this case, G+C ≤ 45% or ≥ 60%) were considered as putative contaminant sequences and removed. On average, 12.10% of the assembled sequences were removed from each genome based on these criteria.

### Generation and assembly of short-read transcriptome data

We generated transcriptome sequence data for the *Symbiodinium* isolates, except for *S. necroappetens* CCMP2469 for which the extraction of total RNAs failed (Additional file 3: Supplementary Table 13). Short-read sequence data (2 × 150 bp reads) were generated using paired-end libraries on the Illumina NovaSeq 6000 platform at the Australian Genome Research Facility (Melbourne). Quality assessment of the raw paired-end data was done with FastQC v0.11.4, and subsequent processing with Trimmomatic v0.35 [76]. To ensure high-quality read data for downstream analyses, the paired-end mode of Trimmomatic was run with the settings: HEADCROP:10 ILLUMINACLIP:[AdapterFile]:2:30:10 CROP:125 SLIDING WINDOW:4:13 MINLEN:50. The surviving read pairs were further trimmed with QUADTrim v2.0.2 (https://bitbucket.org/arobinson/quadtrim) with the flags *-m 2* and *-g* to remove homopolymeric guanine repeats at the end of the reads (a known bias of Illumina NovaSeq 6000 data). Transcriptome assembly was done with Trinity v2.1.1 [84] in de novo and genome-guided modes. De novo transcriptome assembly was done using default parameters and the trimmed read pairs. For genome-guided assembly, high-quality read pairs were aligned to the preliminary de novo genome assembly using Bowtie v2.2.7 [85]. Transcriptomes were then assembled with Trinity in the genome-guided mode using the alignment information, and setting the maximum intron size to 100,000 bp. Both de novo and genome-guided transcriptome assemblies from each sample were used for scaffolding (see above) and gene prediction.

### Generation of full-length transcript data

Full-length transcripts for *S. tridacnidorum* and *S. natans* were generated using the PacBio IsoSeq technology (Additional file 3: Supplementary Table 13). All sequencing was conducted using the PacBio Sequel platform at the Institute for Molecular Bioscience (IMB) Sequencing Facility, The University of Queensland (Brisbane, Australia). Full-length cDNA was first synthesised and amplified using the TeloPrime Full-Length cDNA Amplification Kit (Lexogen) and TeloPrime PCR Add-on Kit (Lexogen) following the protocols provided in the product manuals. One synthesis reaction was performed for each sample using 821 ng from *S. tridacnidorum* and 1.09 μg from *S. natans* of total RNA as starting material. Next, 25 (*S. tridacnidorum*) and 23 (*S. natans*) PCR cycles were carried out for cDNA amplification. PCR products were divided into two fractions, which were purified using 0.5× (for *S. tridacnidorum*) and 1× (for *S. natans*) AMPure PB beads (Pacific Biosciences), and then pooled with equimolar quantities. The recovered 699 ng (*S. tridacnidorum*) and 761 ng (*S. natans*) of cDNA were used for sequencing library preparation with the SMRTbell Template Prep Kit 1.0 (Pacific Biosciences). The cDNA from these libraries were sequenced in two SMRT cells.

To generate the dinoflagellate spliced leader (DinoSL)-specific transcript library, 12 PCR cycles were carried out for both samples using the conserved DinoSL fragment (5′-CCGTAGCCATTTTGGCTCAAG-3′) as forward primer, the TeloPrime PCR 3′-primer as reverse primer and the fraction of full-length cDNA purified with 0.5× (for *S. tridacnidorum*) and 1× (for *S. natans*) AMPure PB beads. The above-described PCR purification and sequencing library preparation methods were used for the DinoSL transcript libraries; cDNA from these libraries was sequenced in one SMRT cell per sample.

Due to the abundance of undesired 5′–5′ and 3′–3′ pairs, and to recover as much transcript evidence as possible for gene prediction, we adopted two approaches for processing IsoSeq data (Additional file 2: Supplementary Figure 13). First, the IsoSeq 3.1 workflow (https://github.com/PacificBiosciences/IsoSeq) was followed. Briefly, circular consensus sequences (CCS) were generated from the subreads of each SMRT cell with ccs v3.1.0 without polishing, and by setting the minimum number of subreads to generate CCS (*--minPasses*) to 1. Removal of primers was done with lima v1.8.0 in the IsoSeq mode, with a subsequent refinement step using IsoSeq v3.1.0. At this stage, the refined full-length transcripts of all SMRT cells (excluding those from the DinoSL library) were combined to be then clustered by similarity and polished with IsoSeq v3.1.0. High- and low-quality transcripts resulting from this approach were further used for gene prediction.

Second, we repeated the IsoSeq workflow with some modifications. We polished the subreads with the Arrow algorithm and used at least three subreads per CCS with ccs v3.1.0 to generate high-accuracy CCS. Primer removal and refinement were done as explained above. The subsequent clustering and polishing steps were skipped. The resulting polished CCS and full-length transcripts were also used for gene prediction. IsoSeq data from the DinoSL library were processed separately following the same two approaches.

### Gene prediction and function annotation

We adopted a comprehensive, customised approach for ab initio gene prediction from dinoflagellate genomes, following earlier studies [32, 33]; the workflow is available at https://github.com/TimothyStephens/Dinoflagellate_Annotation_Workflow. For a detailed schematic overview of this workflow, see Figure S1 in Chen et al. [32]. A de novo repeat library was first derived for the genome assembly using RepeatModeler v1.0.11 (http://www.repeatmasker.org/RepeatModeler/). All repeats (including known repeats in RepeatMasker database release 20180625) were masked using RepeatMasker v4.0.7 (http://www.repeatmasker.org/); masked sequences were used in the subsequent steps for gene prediction. We used scripts available from RepeatMasker to calculate Kimura distances for sequences of each repeat type (*calcDivergenceFromAlign.pl*) and to generate a repeat landscape for each genome (*createRepeatLandscape.pl*).

As direct transcript evidence, we used the de novo and genome-guided transcriptome assemblies from Illumina short-read sequence data. For *S. necroappetens* CCMP2469, we used transcriptome data of the other six *Symbiodinium* isolates for gene prediction, as well as other available transcriptome datasets of *Symbiodinium*: *S. microadriaticum* CassKB8 [86], *S. microadriaticum* CCMP2467 [87], and *S. tridacnidorum* Sh18 [88]. We also combined the *S. microadriaticum* CassKB8 transcriptome data generated here with those from a previous study [86]. For each sample, we concatenated all RNA-Seq transcripts and “cleaned” them using SeqClean (https://sourceforge.net/projects/seqclean/) and the UniVec database build 10.0. For *S. natans* and *S. tridacnidorum*, we also incorporated the PacBio IsoSeq full-length transcript data (above) as evidence to guide gene prediction. We used PASA v2.3.3 [83], customised to recognise dinoflagellate alternative splice donor sites (see above), and TransDecoder v5.2.0 [83] to predict coding sequences (CDS). These CDS were searched (BLASTp, *E* ≤ 10^− 20^) against a protein database that consists of RefSeq proteins (release 88) and a collection of available and predicted (with TransDecoder v5.2.0 [83]) proteins of Symbiodiniaceae (total of 111,591,828 sequences; Additional file 3: Supplementary Table 14). We used the *analyze_blastPlus_topHit_coverage.pl* script from Trinity v2.6.6 [84] to retrieve only those CDS having a hit with > 70% coverage of the database protein sequence (i.e. nearly full-length) in the database for subsequent analyses.

The near full-length gene models were checked for TEs using HHblits v2.0.16 (probability = 80% and *E*-value = 10^− 5^), searching against the JAMg transposon database (https://sourceforge.net/projects/jamg/files/databases/) and TransposonPSI (http://transposonpsi.sourceforge.net/). Gene models containing TEs were removed from the gene set, and redundancy reduction was conducted using cd-hit v4.6 [89, 90] (ID = 75%). The remaining gene models were processed using the *prepare_golden_genes_for_predictors.pl* script from the JAMg pipeline (altered to recognise GA donor splice sites; http://jamg.sourceforge.net/). This script produces a set of “golden genes” that was used as training set for the ab initio gene prediction tools AUGUSTUS v3.3.1 [91] (customised to recognise the non-canonical splice sites of dinoflagellates; https://github.com/chancx/dinoflag-alt-splice) and SNAP v2006-07-28 [92]. Independently, the soft-masked genome sequences were passed to GeneMark-ES v4.32 [93] for unsupervised training and gene prediction. UniProt-Swiss-Prot proteins (downloaded on 27 June 2018) and predicted proteins of Symbiodiniaceae (Additional file 3: Supplementary Table 14) were used to produce a set of gene predictions using MAKER v2.31.10 [94] protein2genome; the custom repeat library was used by RepeatMasker as part of MAKER prediction. A primary set of predicted genes was produced using EvidenceModeler v1.1.1 [95], modified to recognise GA donor splice sites. This package combined the gene predictions from PASA, SNAP, AUGUSTUS, GeneMark-ES and MAKER protein2genome into a single set of evidence-based predictions. The weightings used for the package were as follows: PASA 10, MAKER protein 8, AUGUSTUS 6, SNAP 2 and GeneMark-ES 2. Only gene models with transcript evidence (i.e. predicted by PASA) or supported by at least two ab initio prediction programmes were kept. We assessed completeness by querying the predicted protein sequences in a BLASTp similarity search (*E* ≤ 10^− 5^, ≥ 50% query/target sequence cover) against the 458 core eukaryotic genes from CEGMA [96]. Transcript data support for the predicted genes was determined by BLASTn (*E* ≤ 10^− 5^) similarity search, querying the transcript sequences against the predicted CDS from each genome. Genes for which the transcripts aligned to their CDS with at least 50% of sequence cover and 90% identity were considered as supported by transcript data.

Following Liu et al. [35], functional annotation of the predicted genes was conducted based on sequence similarity searches against known proteins, in which the predicted protein sequences were used as query (BLASTp, *E*  ≤ 10^− 5^, minimum query or target cover of 50%) against the manually curated Swiss-Prot database, and those with no Swiss-Prot hits were subsequently searched against TrEMBL (both databases from UniProt, downloaded 27 June 2018). The best UniProt hit with associated Gene Ontology (GO, http://geneontology.org/) terms was used to annotate the query protein with those GO terms using the UniProt-GOA mapping (downloaded 3 June 2019). Pfam domains [97] were searched in the predicted proteins of all samples using PfamScan [98] (*E* ≤ 0.001) and the Pfam-A database (release 30 August 2018) [97]. Tests for enrichment of Pfam domains were done with one-tailed Fisher’s exact tests, independently for over- and under-represented features; domains with Benjamini and Hochberg [99] adjusted *p* ≤ 0.05 were considered significant. Enrichment of GO terms was performed using the topGO Bioconductor package [100] implemented in R v3.5.1, applying Fisher’s exact test with the ‘elimination’ method to correct for the dependence structure among GO terms. GO terms with a *p* ≤ 0.01 were considered significant.

To assess the potential impact of organellar genome sequences on our analysis, we used available sequences of plastid genomes [101–103] and mitochondrial genes [104–107] from other dinoflagellates as query to search (BLASTn, *E* ≤ 10^− 10^) against the seven de novo assembled genomes from this study. For each genome scaffold that shares significant similarity to known organellar sequences, we assessed if the scaffold contains other protein-coding genes (predicted above) that encode non-organellar functions. We consider those that encode only organellar functions as putative organellar genome sequences.

### Comparison of genome sequences

We compared the genome data of 15 isolates in Order Suessiales: seven for which we generated genome assemblies in this study (Table 1), and as shown in Additional file 3: Supplementary Table 2, eight others represented by *S. microadriaticum* CCMP2467 [87], *S. tridacnidorum* Sh18 [88], *B. minutum* [108], *C. goreaui* [35], *Cladocopium* sp. C92 [88], *F. kawagutii* [35] and *P. glacialis* CCMP1383 and CCMP2088 [33]. Genes were consistently predicted from all genomes using the same workflow above.

Whole-genome sequence alignment was carried out for all possible genome pairs (225 combinations counting each genome as both reference and query) with nucmer v4.0.0 [109], using anchor matches that are unique in the sequences from both reference and query sequences (*--mum*). Here, the similarity between two genomes was assessed based on the proportion of the total bases in the genome sequences of the query that aligned to the reference genome sequences (*Q*) and the average percent identity of one-to-one alignments (i.e. the reciprocal best one-to-one aligned sequences for the implicated region between the query and the reference; *I*). If two genomes are identical, both *Q* and *I* would have a value of 100%. Filtered read pairs (Additional file 3: Supplementary Table 1) from all isolates were aligned to each other’s (and against their own) assembled genome scaffolds using BWA v0.7.13 [110]; mapping rates relative to base quality scores were calculated with SAMStat v1.5.1 [111]. For each possible genome pair, we further assessed sequence similarity of the repeat-masked genome assemblies based on the similarity between their *k*-mers profiles, to capture a comprehensive phylogenetic signal using whole-genome sequences. To determine the appropriate *k*-mer size to use, we extracted and counted *k*-mers using Jellyfish v2.2.6 [81] at multiple *k* values (between 11 and 101, step size = 2); *k* = 21 was found to capture an adequate level of uniqueness among these genomes as inferred based on the proportion of distinct and unique *k*-mers [112] (Additional file 2: Supplementary Figure 14). We then computed pairwise *D*_*2*_^*S*^ distances (*d*) for the 15 isolates following Bernard et al. [30]. The calculated distances were used to build a NJ tree with Neighbor (PHYLIP v3.697) [113] at default settings. For deriving an alignment-free similarity network, pairwise similarity was calculated as 10 − *d* [114]. 

### Analysis of conserved synteny

To assess conserved synteny, we identified collinear syntenic gene blocks common to each genome pair based on the predicted genes and their associated genomic positions. Following Liu et al. [35], we define a syntenic gene block as a region conserved in two genomes in which five or more genes are coded in the same order and orientation. First, we concatenated the sequences of all predicted proteins to conduct all-versus-all BLASTp (*E* ≤ 10^− 5^) searching for similar proteins between each genome pair. The hit pairs were then filtered to include only those where the alignment covered at least half of either the query or the matched protein sequence. Next, we ran *MCScanX* [115] in inter-specific mode (*-b 2*) to identify blocks of at least five genes shared by each genome pair. We independently searched for collinear syntenic blocks within each genome (i.e. duplicated gene blocks). Likewise, we conducted a BLASTp (*E* ≤ 10^−5^) to search for similar proteins within each genome; the hit pairs were filtered to include only those where the alignment covered at least half of either the query or the matched protein sequence. We then ran *MCScanX* in intra-specific mode (*-b 1*).

### Analysis of genic features, gene families and functional enrichment

We examined variation among the predicted genes for all Suessiales isolates with a principal component analysis (PCA) using relevant metrics (Additional file 3: Supplementary Table 4), following Chen et al. [32]. We calculated G+C content in the third position of synonymous codons and effective number of codons used (*Nc*) with CodonW (http://codonw.sourceforge.net/) for complete CDS (defined as those with both start and stop codons) of all isolates. Groups of homologous sequences from all genomes were inferred with OrthoFinder v2.3.1 [116] and considered as gene families. A rooted species tree was inferred using 28,116 families encompassing at least 4 genes from any isolate using STAG [117] and STRIDE [118], following the standard OrthoFinder pipeline. Gene Ontology (GO) enrichment of genes in families core to Symbiodiniaceae and to *Symbiodinium* (defined as those common to all isolates in, and exclusive to, each group) was conducted using the topGO Bioconductor package [100] implemented in R v3.5.1, implementing Fisher’s exact test and the ‘elimination’ method; the GO terms associated to the genes of all isolates surveyed here were used as the background for each comparison. We considered a *p* ≤ 0.01 as significant. The significance of size differences of the gene families shared by *S. tridacnidorum* and *S. natans* was assessed with a two-tailed Fisher’s exact test correcting *p* values for multiple testing [99]; difference in size was considered significant for gene families with adjusted *p* ≤ 0.05. We assessed the relative abundance of gene functions based on the annotated GO terms and protein domains in all predicted genes. For each gene-function feature, we used *Z*-score to compare its relative abundance among the 15 Suessiales genomes, in which *Z* = (*x* − *μ*) / *δ*, where *x* is the relative abundance of a feature in a genome, *μ* is the mean of *x* among all 15 genomes and *δ* is the standard deviation of *μ*. For each genome, *x* for each GO term was calculated relative to the number of genes annotated with the term, and *x* for each protein domain was calculated relative to the total number of domains annotated among all proteins predicted. Hierarchical clustering of *Z*-scores in the heatmap was conducted based on pairwise Spearman’s correlation coefficients using *hclust* implemented in R v4.0.2.

### Analysis of duplicated genes and pseudogenes in *S. tridacnidorum* and *S. natans*

We used the predicted genes and their associated genomic positions to identify potential segmental genome duplications in *S. tridacnidorum* CCMP2592 and *S. natans* CCMP2548, as well as in *P. glacialis* CCMP1383. First, we used BLASTp (*E* ≤ 10^−5^) to search for similar proteins within each genome; the hit pairs were filtered to include only those where the alignment covered at least half of either the query or the matched protein sequence. Next, we ran *MCScanX* [115] in intra-specific mode (*-b 1*) to identify collinear syntenic blocks of at least five genes and genes arranged in tandem within each genome separately.

Identification of genes with DinoSL and pseudogenes was done in a similar way to Song et al. [119]. We queried the original DinoSL sequence (DCCGUAGCCAUUUUGGCUCAAG) [120], excluding the first (ambiguous) position, against the upstream regions (up to 500 bp) of all genes in a BLASTn search, keeping the default values of all alignment parameters but with word size set to 9 (*-word_size* 9). To identify full-length DinoSL sequences in the genome scaffolds, we used the same query above and searched against the assembled genome scaffolds using BLAT [121] (*-tileSize=11 -stepSize=5*). Pseudogenes were identified using tBLASTn, using the predicted protein for each genome as query against the genome sequences, in which regions covered by the predicted genes were masked, as target; in doing so, pseudogenes would not overlap with the predicted gene models. Matched regions with ≥ 75% identity were considered part of pseudogenes and surrounding matching fragments were considered as part of the same pseudogene as long as they were at a maximum distance of 1 kb from another pseudogene fragment and in the same orientation.

## Supplementary Information

**Additional file 1.** Supplementary Note.

**Additional file 2.** Supplementary Figures 1 through 14.

**Additional file 3.** Supplementary Tables 1 through 14.

## Data Availability

All genome and transcriptome sequencing data generated from this study are available at NCBI Short Read Archive (BioProject accession PRJEB34894 [122]). The annotated genomes for all seven *Symbiodinium* isolates are available at NCBI (accessions GCA_905221605.1, GCA_905221615.1, GCA_905221625.1, GCA_905221635.1, GCA_905231905.1, GCA_905231915.1, and GCA_905231925.1). The assembled genomes, predicted gene models and proteins, and putative organellar genome sequences from these isolates are available at 10.14264/f1b3a11 [123].

## References

[1] Baker AC (2003). Flexibility and specificity in coral-algal symbiosis: diversity, ecology, and biogeography of *Symbiodinium*. Annu Rev Ecol Evol Syst.

[2] Muscatine L, Falkowski PG, Porter JW, Dubinsky Z (1984). Fate of photosynthetic fixed carbon in light- and shade-adapted colonies of the symbiotic coral *Stylophora pistillata*. Proc Biol Sci B.

[3] Hughes TP, Barnes ML, Bellwood DR, Cinner JE, Cumming GS, Jackson JBC, Kleypas J, van de Leemput IA, Lough JM, Morrison TH (2017). Coral reefs in the Anthropocene. Nature.

[4] Suggett DJ, Smith DJ (2020). Coral bleaching patterns are the outcome of complex biological and environmental networking. Glob Chang Biol.

[5] Baird AH, Bhagooli R, Ralph PJ, Takahashi S (2009). Coral bleaching: the role of the host. Trends Ecol Evol.

[6] Morris LA, Voolstra CR, Quigley KM, Bourne DG, Bay LK (2019). Nutrient availability and metabolism affect the stability of coral–Symbiodiniaceae symbioses. Trends Microbiol.

[7] Hughes TP, Kerry JT, Álvarez-Noriega M, Álvarez-Romero JG, Anderson KD, Baird AH, Babcock RC, Beger M, Bellwood DR, Berkelmans R (2017). Global warming and recurrent mass bleaching of corals. Nature.

[8] Great Barrier Reef Marine Park Authority, Australian Institute of Marine Science, CSIRO. Reef snapshot: summer 2019–20. Townsville: GBRMPA; 2020.

[9] Bellwood DR, Pratchett MS, Morrison TH, Gurney GG, Hughes TP, Álvarez-Romero JG, Day JC, Grantham R, Grech A, Hoey AS (2019). Coral reef conservation in the Anthropocene: confronting spatial mismatches and prioritizing functions. Biol Conserv.

[10] Vercelloni J, Liquet B, Kennedy EV, González-Rivero M, Caley MJ, Peterson EE, Puotinen M, Hoegh-Guldberg O, Mengersen K (2020). Forecasting intensifying disturbance effects on coral reefs. Glob Chang Biol.

[11] Robbins SJ, Singleton CM, Chan CX, Messer LF, Geers AU, Ying H, Baker A, Bell SC, Morrow KM, Ragan MA (2019). A genomic view of the reef-building coral *Porites lutea* and its microbial symbionts. Nat Microbiol.

[12] Wright BR, Farquharson KA, McLennan EA, Belov K, Hogg CJ, Grueber CE. A demonstration of conservation genomics for threatened species management. Mol Ecol Resour. 2020;20(6):1526⁠–41. 10.1111/1755-0998.13211 32562371

[13] LaJeunesse TC, Lambert G, Andersen RA, Coffroth MA, Galbraith DW (2005). *Symbiodinium* (Pyrrhophyta) genome sizes (DNA content) are smallest among dinoflagellates. J Phycol.

[14] Saad OS, Lin X, Ng TY, Li L, Ang P, Lin S (2020). Genome size, rDNA copy, and qPCR assays for Symbiodiniaceae. Front Microbiol.

[15] Lin S (2011). Genomic understanding of dinoflagellates. Res Microbiol.

[16] Wisecaver JH, Hackett JD (2011). Dinoflagellate genome evolution. Annu Rev Microbiol.

[17] Correa A, Baker AC (2009). Understanding diversity in coral-algal symbiosis: a cluster-based approach to interpreting fine-scale genetic variation in the genus *Symbiodinium*. Coral Reefs.

[18] Forsman ZH, Ritson-Williams R, Tisthammer KH, Knapp ISS, Toonen RJ (2020). Host-symbiont coevolution, cryptic structure, and bleaching susceptibility, in a coral species complex (Scleractinia; Poritidae). Sci Rep.

[19] Kimura M (1968). Evolutionary rate at the molecular level. Nature.

[20] González-Pech RA, Bhattacharya D, Ragan MA, Chan CX (2019). Genome evolution of coral reef symbionts as intracellular residents. Trends Ecol Evol.

[21] Moran NA, Plague GR (2004). Genomic changes following host restriction in bacteria. Curr Opin Genet Dev.

[22] McCutcheon JP, Moran NA (2012). Extreme genome reduction in symbiotic bacteria. Nat Rev Microbiol.

[23] Quigley K, Bay LK, Willis B (2017). Temperature and water quality-related patterns in sediment-associated *Symbiodinium* communities impact symbiont uptake and fitness of juveniles in the genus *Acropora*. Front Mar Sci.

[24] Nitschke MR, Davy SK, Cribb TH, Ward S (2015). The effect of elevated temperature and substrate on free-living *Symbiodinium* cultures. Coral Reefs.

[25] Murray S, Flo Jorgensen M, Ho SY, Patterson DJ, Jermiin LS (2005). Improving the analysis of dinoflagellate phylogeny based on rDNA. Protist.

[26] LaJeunesse TC, Parkinson JE, Gabrielson PW, Jeong HJ, Reimer JD, Voolstra CR, Santos SR (2018). Systematic revision of Symbiodiniaceae highlights the antiquity and diversity of coral endosymbionts. Curr Biol.

[27] Montresor M, Procaccini G, Stoecker DK (1999). *Polarella glacialis*, gen. nov., sp. nov. (Dinophyceae): Suessiaceae are still alive!. J Phycol.

[28] Pandeirada MS, Craveiro SC, Daugbjerg N, Moestrup Ø, Calado AJ (2021). Fine-structural characterization and phylogeny of *Sphaerodinium* (Suessiales, Dinophyceae), with the description of an unusual type of freshwater dinoflagellate cyst. Eur J Protistol.

[29] Bernard G, Chan CX, Chan YB, Chua XY, Cong Y, Hogan JM, Maetschke SR, Ragan MA (2019). Alignment-free inference of hierarchical and reticulate phylogenomic relationships. Brief Bioinform.

[30] Bernard G, Greenfield P, Ragan MA, Chan CX. *k*-mer similarity, networks of microbial genomes, and taxonomic rank. mSystems. 2018;3(6):e00257–18. 10.1128/mSystems.00257-18 PMC6247013 30505941

[31] Hansen G, Daugbjerg N (2009). *Symbiodinium natans* sp. nov.: a "free-living" dinoflagellate from Tenerife (Northeast-Atlantic Ocean). J Phycol.

[32] Chen Y, González-Pech RA, Stephens TG, Bhattacharya D, Chan CX (2020). Evidence that inconsistent gene prediction can mislead analysis of dinoflagellate genomes. J Phycol.

[33] Stephens TG, González-Pech RA, Cheng Y, Mohamed AR, Burt DW, Bhattacharya D, Ragan MA, Chan CX (2020). Genomes of the dinoflagellate *Polarella glacialis* encode tandemly repeated single-exon genes with adaptive functions. BMC Biol.

[34] Kimura M (1980). A simple method for estimating evolutionary rates of base substitutions through comparative studies of nucleotide sequences. J Mol Evol.

[35] Liu H, Stephens TG, González-Pech RA, Beltran VH, Lapeyre B, Bongaerts P, Cooke I, Aranda M, Bourne DG, Forêt S (2018). *Symbiodinium* genomes reveal adaptive evolution of functions related to coral-dinoflagellate symbiosis. Commun Biol.

[36] González-Pech RA, Ragan MA, Chan CX (2017). Signatures of adaptation and symbiosis in genomes and transcriptomes of *Symbiodinium*. Sci Rep.

[37] Stephens TG, Ragan MA, Bhattacharya D, Chan CX (2018). Core genes in diverse dinoflagellate lineages include a wealth of conserved dark genes with unknown functions. Sci Rep.

[38] Jaeckisch N, Yang I, Wohlrab S, Glöckner G, Kroymann J, Vogel H, Cembella A, John U (2011). Comparative genomic and transcriptomic characterization of the toxigenic marine dinoflagellate *Alexandrium ostenfeldii*. PLoS One.

[39] van Baren MJ, Bachy C, Reistetter EN, Purvine SO, Grimwood J, Sudek S, Yu H, Poirier C, Deerinck TJ, Kuo A (2016). Evidence-based green algal genomics reveals marine diversity and ancestral characteristics of land plants. BMC Genomics.

[40] Read BA, Kegel J, Klute MJ, Kuo A, Lefebvre SC, Maumus F, Mayer C, Miller J, Monier A, Salamov A (2013). Pan genome of the phytoplankton *Emiliania* underpins its global distribution. Nature.

[41] Mohamed AR, Andrade N, Moya A, Chan CX, Negri AP, Bourne DG, Ying H, Ball EE, Miller DJ (2020). Dual RNA-sequencing analyses of a coral and its native symbiont during the establishment of symbiosis. Mol Ecol.

[42] Weis VM (2019). Cell biology of coral symbiosis: foundational study can inform solutions to the coral reef crisis. Integr Comp Biol.

[43] Li C, Wong JTY (2019). DNA damage response pathways in dinoflagellates. Microorganisms.

[44] Chi J, Parrow MW, Dunthorn M (2014). Cryptic sex in *Symbiodinium* (Alveolata, Dinoflagellata) is supported by an inventory of meiotic genes. J Eukaryot Microbiol.

[45] Shah S, Chen Y, Bhattacharya D, Chan CX (2020). Sex in Symbiodiniaceae dinoflagellates: genomic evidence for independent loss of the canonical synaptonemal complex. Sci Rep.

[46] Baillie B, Belda-Baillie C, Silvestre V, Sison M, Gomez A, Gomez E, Monje V (2000). Genetic variation in *Symbiodinium* isolates from giant clams based on random-amplified-polymorphic DNA (RAPD) patterns. Mar Biol.

[47] Baillie B, Monje V, Silvestre V, Sison M, Belda-Baillie C (1998). Allozyme electrophoresis as a tool for distinguishing different zooxanthellae symbiotic with giant clams. Proc Biol Sci B.

[48] LaJeunesse T (2002). Diversity and community structure of symbiotic dinoflagellates from Caribbean coral reefs. Mar Biol.

[49] Pettay DT, LaJeunesse TC (2013). Long-range dispersal and high-latitude environments influence the population structure of a “stress-tolerant” dinoflagellate endosymbiont. PLoS One.

[50] Thornhill DJ, Lewis AM, Wham DC, LaJeunesse TC (2014). Host-specialist lineages dominate the adaptive radiation of reef coral endosymbionts. Evolution.

[51] Figueroa RI, Dapena C, Bravo I, Cuadrado A (2015). The hidden sexuality of *Alexandrium minutum*: an example of overlooked sex in dinoflagellates. PLoS One.

[52] Lee SY, Jeong HJ, Kang NS, Jang TY, Jang SH, Lajeunesse TC (2015). *Symbiodinium tridacnidorum* sp. nov., a dinoflagellate common to indo-Pacific giant clams, and a revised morphological description of *Symbiodinium microadriaticum* Freudenthal, emended Trench & Blank. Eur J Phycol.

[53] Carlos AA, Baillie BK, Kawachi M, Maruyama T (1999). Phylogenetic position of *Symbiodinium* (Dinophyceae) isolates from tridacnids (Bivalvia), cardiids (Bivalvia), a sponge (Porifera), a soft coral (Anthozoa), and a free-living strain. J Phycol.

[54] Lin S, Cheng S, Song B, Zhong X, Lin X, Li W, Li L, Zhang Y, Zhang H, Ji Z (2015). The *Symbiodinium kawagutii* genome illuminates dinoflagellate gene expression and coral symbiosis. Science.

[55] de Mendoza A, Bonnet A, Vargas-Landin DB, Ji N, Hong F, Yang F, Li L, Hori K, Pflueger J, Buckberry S (2018). Recurrent acquisition of cytosine methyltransferases into eukaryotic retrotransposons. Nat Commun.

[56] Ying H, Cooke I, Sprungala S, Wang W, Hayward DC, Tang Y, Huttley G, Ball EE, Forêt S, Miller DJ (2018). Comparative genomics reveals the distinct evolutionary trajectories of the robust and complex coral lineages. Genome Biol.

[57] Saliba KJ, Martin RE, Bröer A, Henry RI, Siobhan McCarthy C, Downie MJ, Allen RJW, Mullin KA, McFadden GI, Bröer S (2006). Sodium-dependent uptake of inorganic phosphate by the intracellular malaria parasite. Nature.

[58] Zhang H, Campbell DA, Sturm NR, Lin S (2009). Dinoflagellate spliced leader RNA genes display a variety of sequences and genomic arrangements. Mol Biol Evol.

[59] Bachvaroff TR, Place AR (2008). From stop to start: tandem gene arrangement, copy number and *trans*-splicing sites in the dinoflagellate *Amphidinium carterae*. PLoS One.

[60] Le QH, Markovic P, Hastings JW, Jovine RVM, Morse D (1997). Structure and organization of the peridinin-chlorophyll a-binding protein gene in *Gonyaulax polyedra*. Mol Gen Genet.

[61] Prince VE, Pickett FB (2002). Splitting pairs: the diverging fates of duplicated genes. Nat Rev Genet.

[62] Liew YJ, Li Y, Baumgarten S, Voolstra CR, Aranda M (2017). Condition-specific RNA editing in the coral symbiont *Symbiodinium microadriaticum*. PLoS Genet.

[63] John U, Lu Y, Wohlrab S, Groth M, Janouskovec J, Kohli GS, Mark FC, Bickmeyer U, Farhat S, Felder M (2019). An aerobic eukaryotic parasite with functional mitochondria that likely lacks a mitochondrial genome. Sci Adv.

[64] Mock T, Otillar RP, Strauss J, McMullan M, Paajanen P, Schmutz J, Salamov A, Sanges R, Toseland A, Ward BJ (2017). Evolutionary genomics of the cold-adapted diatom *Fragilariopsis cylindrus*. Nature.

[65] Brian JI, Davy SK, Wilkinson SP (2019). Multi-gene incongruence consistent with hybridisation in *Cladocopium* (Symbiodiniaceae), an ecologically important genus of coral reef symbionts. PeerJ.

[66] Cordaux R, Batzer MA (2009). The impact of retrotransposons on human genome evolution. Nat Rev Genet.

[67] Quadrana L, Etcheverry M, Gilly A, Caillieux E, Madoui M-A, Guy J, Bortolini Silveira A, Engelen S, Baillet V, Wincker P (2019). Transposition favors the generation of large effect mutations that may facilitate rapid adaption. Nat Commun.

[68] Pochon X, Montoya-Burgos JI, Stadelmann B, Pawlowski J (2006). Molecular phylogeny, evolutionary rates, and divergence timing of the symbiotic dinoflagellate genus *Symbiodinium*. Mol Phylogenet Evol.

[69] Stat M, Carter D, Hoegh-Guldberg O (2006). The evolutionary history of *Symbiodinium* and scleractinian hosts—symbiosis, diversity, and the effect of climate change. Perspect Plant Ecol.

[70] Bravo I, Figueroa RI (2014). Towards an ecological understanding of dinoflagellate cyst functions. Microorganisms.

[71] John U, Fensome RA, Medlin LK (2003). The application of a molecular clock based on molecular sequences and the fossil record to explain biogeographic distributions within the Alexandrium tamarense "species complex" (Dinophyceae). Mol Biol Evol.

[72] Simpson C, Kiessling W, Mewis H, Baron-Szabo RC, Muller J (2011). Evolutionary diversification of reef corals: a comparison of the molecular and fossil records. Evolution.

[73] LaJeunesse TC, Pettay DT, Sampayo EM, Phongsuwan N, Brown B, Obura DO, Hoegh-Guldberg O, Fitt WK (2010). Long-standing environmental conditions, geographic isolation and host–symbiont specificity influence the relative ecological dominance and genetic diversification of coral endosymbionts in the genus *Symbiodinium*. J Biogeogr.

[74] LaJeunesse TC (2005). “Species” radiations of symbiotic dinoflagellates in the Atlantic and indo-Pacific since the Miocene-Pliocene transition. Mol Biol Evol.

[75] Thornhill DJ, Howells EJ, Wham DC, Steury TD, Santos SR (2017). Population genetics of reef coral endosymbionts (*Symbiodinium*, Dinophyceae). Mol Ecol.

[76] Bolger AM, Lohse M, Usadel B (2014). Trimmomatic: a flexible trimmer for Illumina sequence data. Bioinformatics.

[77] Magoč T, Salzberg SL (2011). FLASH: fast length adjustment of short reads to improve genome assemblies. Bioinformatics.

[78] O’Connell J, Schulz-Trieglaff O, Carlson E, Hims MM, Gormley NA, Cox AJ (2015). NxTrim: optimized trimming of Illumina mate pair reads. Bioinformatics.

[79] Xue W, Li J-T, Zhu Y-P, Hou G-Y, Kong X-F, Kuang Y-Y, Sun X-W (2013). L_RNA_scaffolder: scaffolding genomes with transcripts. BMC Genomics.

[80] Zimin AV, Puiu D, Luo M-C, Zhu T, Koren S, Marçais G, Yorke JA, Dvořák J, Salzberg SL (2017). Hybrid assembly of the large and highly repetitive genome of *Aegilops tauschii*, a progenitor of bread wheat, with the MaSuRCA mega-reads algorithm. Genome Res.

[81] Marçais G, Kingsford C (2011). A fast, lock-free approach for efficient parallel counting of occurrences of *k*-mers. Bioinformatics.

[82] Ranallo-Benavidez TR, Jaron KS, Schatz MC (2020). GenomeScope 2.0 and Smudgeplot for reference-free profiling of polyploid genomes. Nat Commun.

[83] Haas BJ, Delcher AL, Mount SM, Wortman JR, Smith RK, Hannick LI, Maiti R, Ronning CM, Rusch DB, Town CD (2003). Improving the *Arabidopsis* genome annotation using maximal transcript alignment assemblies. Nucleic Acids Res.

[84] Haas BJ, Papanicolaou A, Yassour M, Grabherr M, Blood PD, Bowden J, Couger MB, Eccles D, Li B, Lieber M (2013). *De novo* transcript sequence reconstruction from RNA-seq using the trinity platform for reference generation and analysis. Nat Protoc.

[85] Langmead B, Salzberg SL (2012). Fast gapped-read alignment with Bowtie 2. Nat Methods.

[86] Bayer T, Aranda M, Sunagawa S, Yum LK, DeSalvo MK, Lindquist E, Coffroth MA, Voolstra CR, Medina M (2012). *Symbiodinium* transcriptomes: genome insights into the dinoflagellate symbionts of reef-building corals. PLoS One.

[87] Aranda M, Li Y, Liew YJ, Baumgarten S, Simakov O, Wilson MC, Piel J, Ashoor H, Bougouffa S, Bajic VB (2016). Genomes of coral dinoflagellate symbionts highlight evolutionary adaptations conducive to a symbiotic lifestyle. Sci Rep.

[88] Shoguchi E, Beedessee G, Tada I, Hisata K, Kawashima T, Takeuchi T, Arakaki N, Fujie M, Koyanagi R, Roy MC (2018). Two divergent *Symbiodinium* genomes reveal conservation of a gene cluster for sunscreen biosynthesis and recently lost genes. BMC Genomics.

[89] Fu L, Niu B, Zhu Z, Wu S, Li W (2012). CD-HIT: accelerated for clustering the next-generation sequencing data. Bioinformatics.

[90] Li W, Godzik A (2006). Cd-hit: a fast program for clustering and comparing large sets of protein or nucleotide sequences. Bioinformatics.

[91] Stanke M, Keller O, Gunduz I, Hayes A, Waack S, Morgenstern B (2006). AUGUSTUS: *ab initio* prediction of alternative transcripts. Nucleic Acids Res.

[92] Korf I (2004). Gene finding in novel genomes. BMC Bioinformatics.

[93] Lomsadze A, Ter-Hovhannisyan V, Chernoff YO, Borodovsky M (2005). Gene identification in novel eukaryotic genomes by self-training algorithm. Nucleic Acids Res.

[94] Holt C, Yandell M (2011). MAKER2: an annotation pipeline and genome-database management tool for second-generation genome projects. BMC Bioinformatics.

[95] Haas BJ, Salzberg SL, Zhu W, Pertea M, Allen JE, Orvis J, White O, Buell CR, Wortman JR (2008). Automated eukaryotic gene structure annotation using EVidenceModeler and the program to assemble spliced alignments. Genome Biol.

[96] Parra G, Bradnam K, Korf I (2007). CEGMA: a pipeline to accurately annotate core genes in eukaryotic genomes. Bioinformatics.

[97] Finn RD, Coggill P, Eberhardt RY, Eddy SR, Mistry J, Mitchell AL, Potter SC, Punta M, Qureshi M, Sangrador-Vegas A (2015). The Pfam protein families database: towards a more sustainable future. Nucleic Acids Res.

[98] Li W, Cowley A, Uludag M, Gur T, McWilliam H, Squizzato S, Park YM, Buso N, Lopez R (2015). The EMBL-EBI bioinformatics web and programmatic tools framework. Nucleic Acids Res.

[99] Benjamini Y, Hochberg Y (1995). Controlling the false discovery rate: a practical and powerful approach to multiple testing. J R Stat Soc B.

[100] Alexa A, Rahnenfuhrer J. topGO: enrichment analysis for Gene Ontology. 2010. R package

[101] Zhang Z, Green B, Cavalier-Smith T (1999). Single gene circles in dinoflagellate chloroplast genomes. Nature.

[102] Barbrook AC, Voolstra CR, Howe CJ (2014). The chloroplast genome of a *Symbiodinium* sp. clade C3 isolate. Protist.

[103] Mungpakdee S, Shinzato C, Takeuchi T, Kawashima T, Koyanagi R, Hisata K, Tanaka M, Goto H, Fujie M, Lin S (2014). Massive gene transfer and extensive RNA editing of a symbiotic dinoflagellate plastid genome. Genome Biol Evol.

[104] Jackson CJ, Norman JE, Schnare MN, Gray MW, Keeling PJ, Waller RF (2007). Broad genomic and transcriptional analysis reveals a highly derived genome in dinoflagellate mitochondria. BMC Biol.

[105] Kamikawa R, Nishimura H, Sako Y (2009). Analysis of the mitochondrial genome, transcripts, and electron transport activity in the dinoflagellate *Alexandrium catenella* (Gonyaulacales, Dinophyceae). Phycol Res.

[106] Shoguchi E, Shinzato C, Hisata K, Satoh N, Mungpakdee S (2015). The large mitochondrial genome of *Symbiodinium minutum* reveals conserved noncoding sequences between dinoflagellates and apicomplexans. Genome Biol Evol.

[107] Hume BC, Voolstra CR, Arif C, D'Angelo C, Burt JA, Eyal G, Loya Y, Wiedenmann J (2016). Ancestral genetic diversity associated with the rapid spread of stress-tolerant coral symbionts in response to Holocene climate change. Proc Natl Acad Sci U S A.

[108] Shoguchi E, Shinzato C, Kawashima T, Gyoja F, Mungpakdee S, Koyanagi R, Takeuchi T, Hisata K, Tanaka M, Fujiwara M (2013). Draft assembly of the *Symbiodinium minutum* nuclear genome reveals dinoflagellate gene structure. Curr Biol.

[109] Marçais G, Delcher AL, Phillippy AM, Coston R, Salzberg SL, Zimin A (2018). MUMmer4: a fast and versatile genome alignment system. PLoS Comput Biol.

[110] Li H, Durbin R (2010). Fast and accurate long-read alignment with Burrows–Wheeler transform. Bioinformatics.

[111] Lassmann T, Hayashizaki Y, Daub CO (2011). SAMStat: monitoring biases in next generation sequencing data. Bioinformatics.

[112] Greenfield P, Roehm U (2013). Answering biological questions by querying *k*-mer databases. Concurr Comp.

[113] Felsenstein J. Phylogenies Inference Package (PHYLIP) version 3.69. Seattle: Department of Genome Sciences, University of Washington; 2008.

[114] Bernard G, Ragan MA, Chan CX (2016). Recapitulating phylogenies using *k*-mers: from trees to networks [version 2; peer review: 2 approved]. F1000Research.

[115] Wang Y, Tang H, DeBarry JD, Tan X, Li J, Wang X, Lee T-H, Jin H, Marler B, Guo H (2012). *MCScanX*: a toolkit for detection and evolutionary analysis of gene synteny and collinearity. Nucleic Acids Res.

[116] Emms DM, Kelly S (2019). OrthoFinder: phylogenetic orthology inference for comparative genomics. Genome Biol.

[117] Emms DM, Kelly S. STAG: Species Tree Inference from All Genes. bioRxiv. 2018, 267914.

[118] Emms DM, Kelly S (2017). STRIDE: species tree root inference from gene duplication events. Mol Biol Evol.

[119] Song B, Morse D, Song Y, Fu Y, Lin X, Wang W, Cheng S, Chen W, Liu X, Lin S (2017). Comparative genomics reveals two major bouts of gene retroposition coinciding with crucial periods of *Symbiodinium* evolution. Genome Biol Evol.

[120] Zhang H, Hou Y, Miranda L, Campbell DA, Sturm NR, Gaasterland T, Lin S (2007). Spliced leader RNA trans-splicing in dinoflagellates. Proc Natl Acad Sci U S A.

[121] Kent WJ (2002). BLAT—the BLAST-like alignment tool. Genome Res.

[122] Genomic and Transcriptomic study of *Symbiodinium* Isolates. NCBI. 2020. https://www.ncbi.nlm.nih.gov/bioproject/PRJEB34894.

[123] González-Pech RA, Ragan MA, Bhattacharya D, Chan CX. Genome assemblies and the associated annotations for seven *Symbiodinium* isolates: The University of Queensland; 2021. Data Collection. 10.14264/f1b3a11

