# Supplementary material for: Comparison of 15 dinoflagellate genomes reveals extensive sequence and structural divergence in family Symbiodiniaceae and genus Symbiodinium
Source: BMC Biol. 2021 Apr 13;19:73. doi: 10.1186/s12915-021-00994-6 (PMC8045281; doi:10.1186/s12915-021-00994-6)
Supplement: Supplementary file 1 — Additional file 1. Supplementary Note. [file 12915_2021_994_MOESM1_ESM.pdf]

Supplementary Note

Genomes of Symbiodinium tridacnidorum and Symbiodinium natans are highly divergent

The genomes of *S. tridacnidorum* and *S. natans* are highly dissimilar from one another (Fig. 6a). According to our analysis of enriched gene functions in *S. tridacnidorum* relative to *S. natans* based on annotated GO terms, methylation and the biosynthesis of histidine and peptidoglycan were among the most significant (Additional File 3: Supplementary Table 7). The enrichment of methylation is not surprising because retrotransposons of Symbiodiniaceae are known to have acquired methyltransferase domains, likely contributing to the hypermethylated nuclear genomes of these dinoflagellates [55]. The link between the extent of methylation in symbiodiniacean genomes and its representation among predicted genes can be further assessed using methylation sequencing. Although some corals can synthesise histidine *de novo*, metazoans generally lack this capacity [56]. The enrichment of histidine biosynthesis in *S. tridacnidorum* may be a result of host-symbiont coevolution or, alternatively, may explain why this species is a preferred symbiont over others (e.g. *S. natans*). Biosynthesis of peptidoglycans is also important for symbiosis because these molecules, on the cell surface of Symbiodiniaceae, interact with host lectins as part of the symbiont recognition process [42].

On the other hand, *S. natans* displays a wider range of enriched functions related to cellular processes (Additional File 3: Supplementary Table 7), as expected for free-living Symbiodiniaceae [20]. One of the most significantly overrepresented gene functions is the transmembrane transport of sodium. Whereas this function is likely related to pH (osmotic) homeostasis with the extracellular environment, the occurrence of a sodium:phosphate symporter (PF02690) in tandem, exclusive to *S. natans*, and the abundance of a sodium:chloride symporter (PF00209) among the RT-genes (Additional File 3: Supplementary Table 8) suggest that *S. natans* makes use of the Na^+ differential gradient (caused by the higher Na^+ concentration in seawater) for nutrient uptake in a similar fashion to the assimilation of inorganic phosphate by the malaria parasite (*Plasmodium falciparum*) in the Na^+-rich cytosol of the host's erythrocytes [57].

Retroposition, retrotransposition and gene duplication in S. tridacnidorum versus S. natans

Both retroposition and retrotransposition have been reported to contribute to gene-family expansion in Symbiodiniaceae [54]. Protein domains with functions related to retrotransposition were overrepresented in gene products of *S. tridacnidorum* relative to those of *S. natans* (Additional File 3: Supplementary Table 9). However, reverse transcriptase domains (PF00078 and PF07727) are abundant in both; they were found in 1313 predicted proteins in *S. tridacnidorum* and 591 in *S. natans*. Retrotransposons can accelerate mutation rate [67] and alter the architecture of genes in their flanking regions [66], and may explain the emergence of genes coding for reverse transcriptase domains (RT-genes) in these genomes.

Other domains found in these proteins are involved in diverse cellular processes including ubiquitin-mediated proteolysis, DNA methylation, transmembrane transport, and photosynthesis (Additional File 3: Supplementary Table 8).

Are features underpinning genome divergence in Symbiodiniaceae ancestral or derived?

To assess whether the genome features found in *S. tridacnidorum* (and their putative association with symbiotic lifestyle) were ancestral or derived relative to *S. natans*, we compared the genome sequences from both species with those from the outgroup *Polarella glacialis* CCMP1383 [33], a psychrophilic free-living species closely related to Symbiodiniaceae (also in Order Suessiales). A greater genome sequence proportion of *S. natans* (3.38%) than that of *S. tridacnidorum* (0.85%) aligned to the *P. glacialis* genome assembly. Interestingly, the aligned regions in both cases implicate only ~5 Mbp (~0.18%) of the *P. glacialis* genome sequence. This observation is likely due to duplicated genome regions of *S. natans* that have remained highly conserved. Similarly, the average percent identity of the best-matching sequences between any of the two *Symbiodinium* genomes against *P. glacialis* is very similar (i.e. 92.13% and 92.56% for *S. tridacnidorum* and *S. natans*, respectively). Based on our analysis of repeat content in these three genomes, LTR retrotransposons are evidently more prominent in *P. glacialis*; these LTRs are more diverged (Kimura distances 3-8) [33] than those in the two *Symbiodinium* (Kimura distances < 5; Fig. 3b), indicating an independent, more-ancient burst of these elements in *P. glacialis*.

Because most other dinoflagellate lineages external to Symbiodiniaceae are free-living, the symbiotic lifestyle can be assumed to be a derived trait in *Symbiodinium*. Under this assumption, the genome proportion spanned by TEs and duplicated genes in the free-living *S. natans* is expected to be similar to (if not smaller than) that of *P. glacialis* [33]. However, we found the proportion of tandem repeated genes and LINEs to be larger in *S. natans* than in *P. glacialis* (Additional File 2: Supplementary Fig. 11). prompting two possible explanations. First, the pervasive simple repeats in the *P. glacialis* genome [33], either independently expanded along this lineage or an ancestral trait in Suessiales, drastically diminish the proportion of genic regions in the genome. Second, the free-living lifestyle of *S. natans* may be a derived trait in *Symbiodinium* following an earlier symbiotic phase in the evolutionary history of this species. Symbiotic Suessiales taxa sister to Symbiodiniaceae have also been described, e.g. *Pelagodinium béii* (genome data not available). Under this notion, the robust placement of the free-living *S. natans* and *S. pilosum* as separate lineages in the *Symbiodinium* phylogeny [26] (and not in a monophyletic group), suggests that the free-living lifestyle in these two species may have arisen independently. Additional high-quality genome data from free-living and symbiotic taxa are required to further elucidate the impact of evolutionary transition(s) between free-living and symbiotic lifestyles on evolution of Symbiodiniaceae.
