# Supplementary material for: Comparison of 15 dinoflagellate genomes reveals extensive sequence and structural divergence in family Symbiodiniaceae and genus Symbiodinium
Source: BMC Biol. 2021 Apr 13;19:73. doi: 10.1186/s12915-021-00994-6 (PMC8045281; doi:10.1186/s12915-021-00994-6)
Supplement: Supplementary file 2 — Additional file 2. Supplementary Figures 1 through 14. [file 12915_2021_994_MOESM2_ESM.pdf]

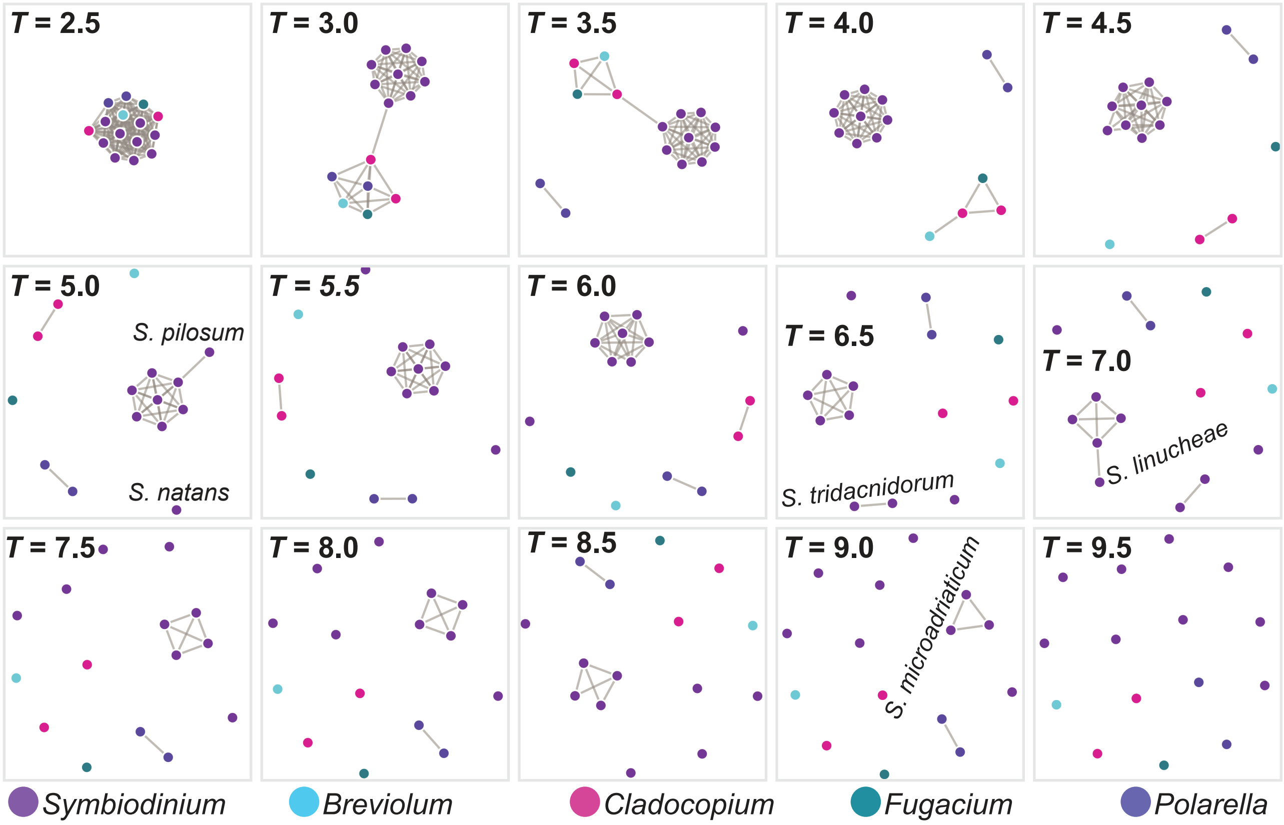

Supplementary Fig. 1. Phylogenetic network of Suessiales genomes based on alignment-free analysis of 21-mers. Similarity network constructed based on shared 21-mers among the genome sequences of the Suessiales isolates. Each panel shows the network at a similarity threshold (*T*), at edges (connections) with a similarity value below the threshold are discarded; see Bernard *et al.* [30] for more detail. Data points representing each dataset are coloured following the bottom legend. *S. microadriaticum* isolates are the last *Symbiodinium* clique at $T = 9.0$.

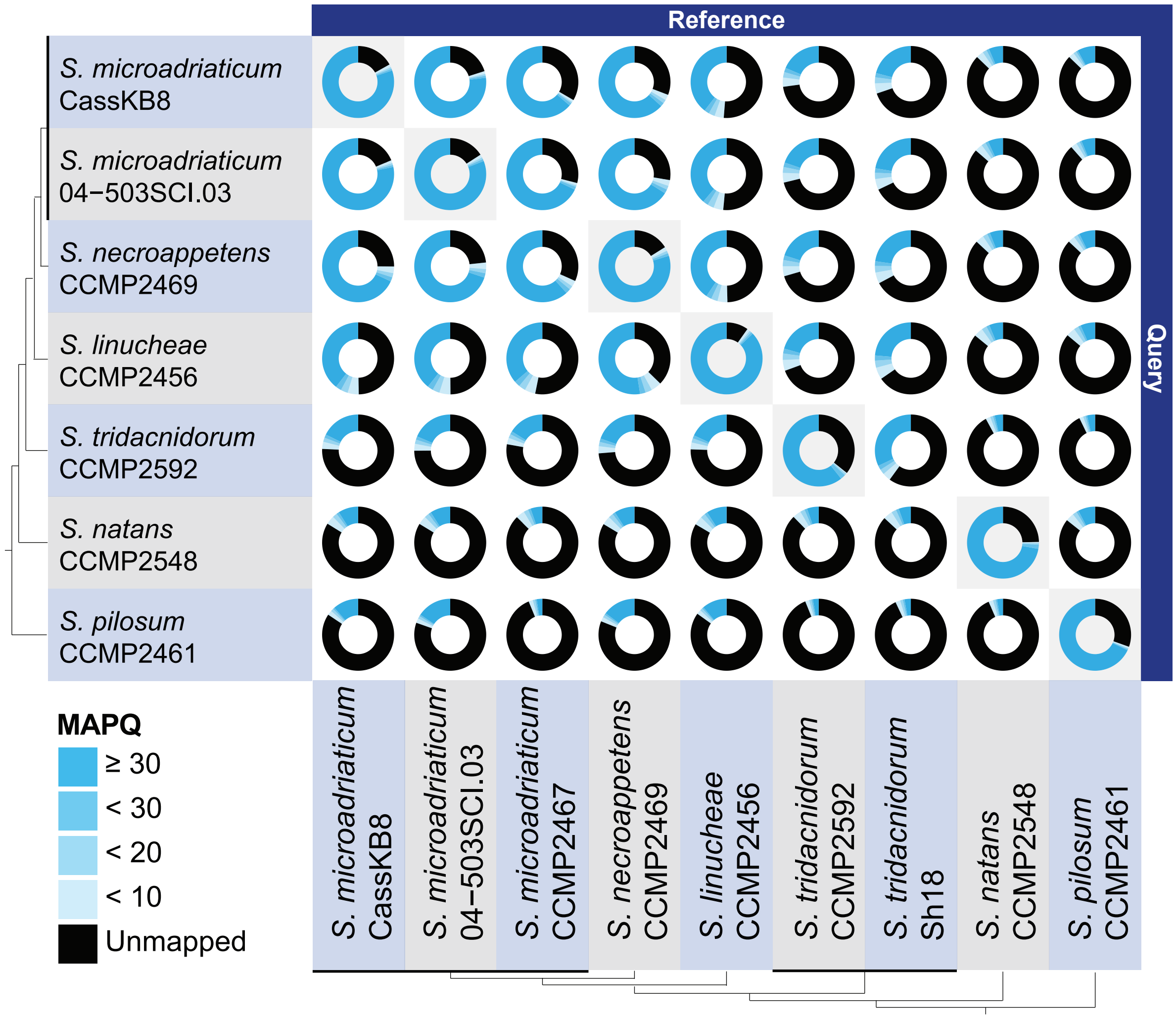

Supplementary Fig. 2. Mapping rate of filtered paired reads that we generated for each *Symbiodinium* isolate against the assembled genomes of itself (grey background) and of all other *Symbiodinium* isolates. The tree topologies on the left and bottom indicate the known phylogenetic relationship [26] among the isolates.

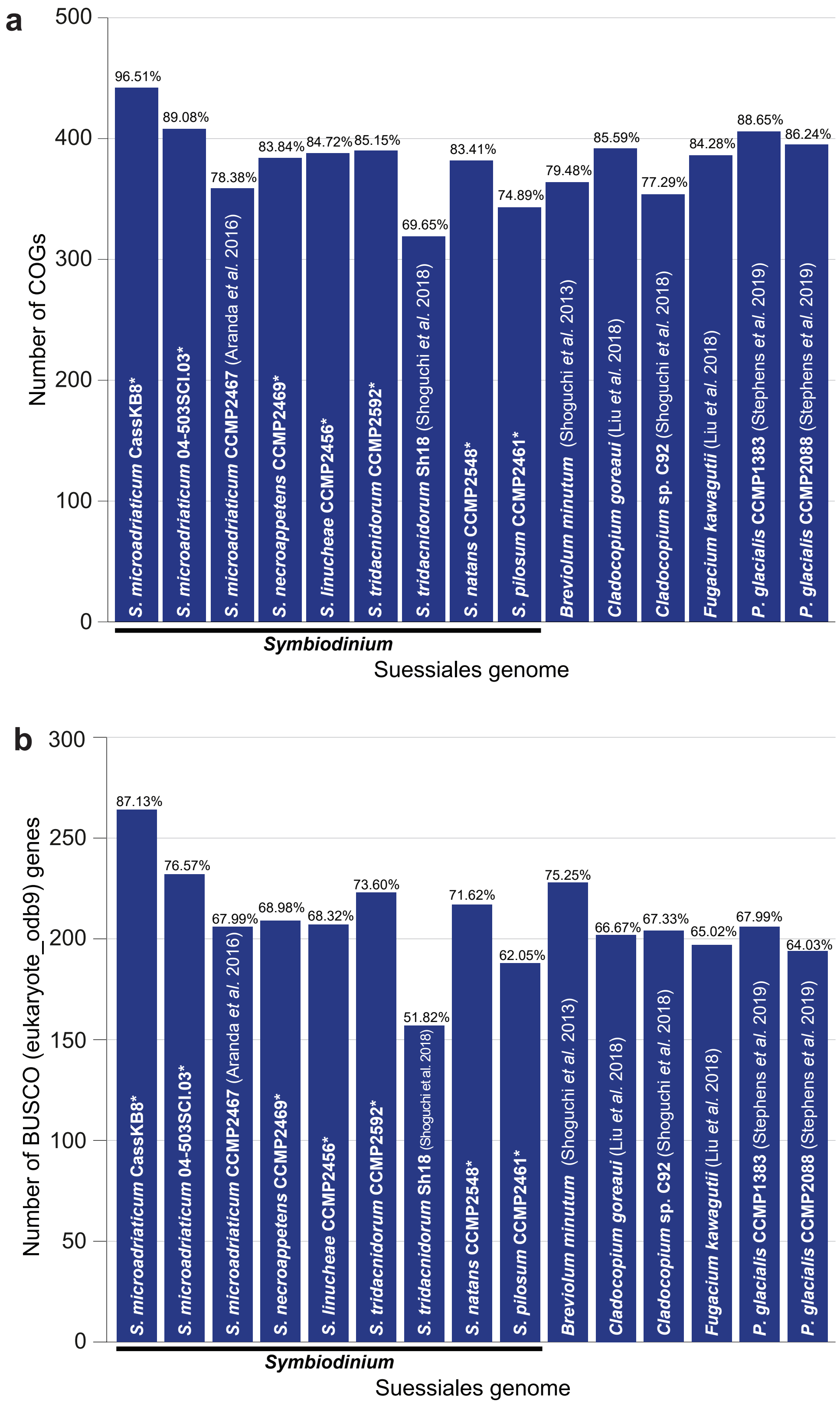

Supplementary Fig. 3. Recovery of core conserved eukaryote genes among the predicted genes in each genome, based on (a) 458 CEGMA Clusters of Orthologous Groups, and (b) 303 BUSCO eukaryote_odb09 genes. Isolates for which genome data were generated in this study are indicated with an asterisk. The percentage of recovered genes is shown for each bar.

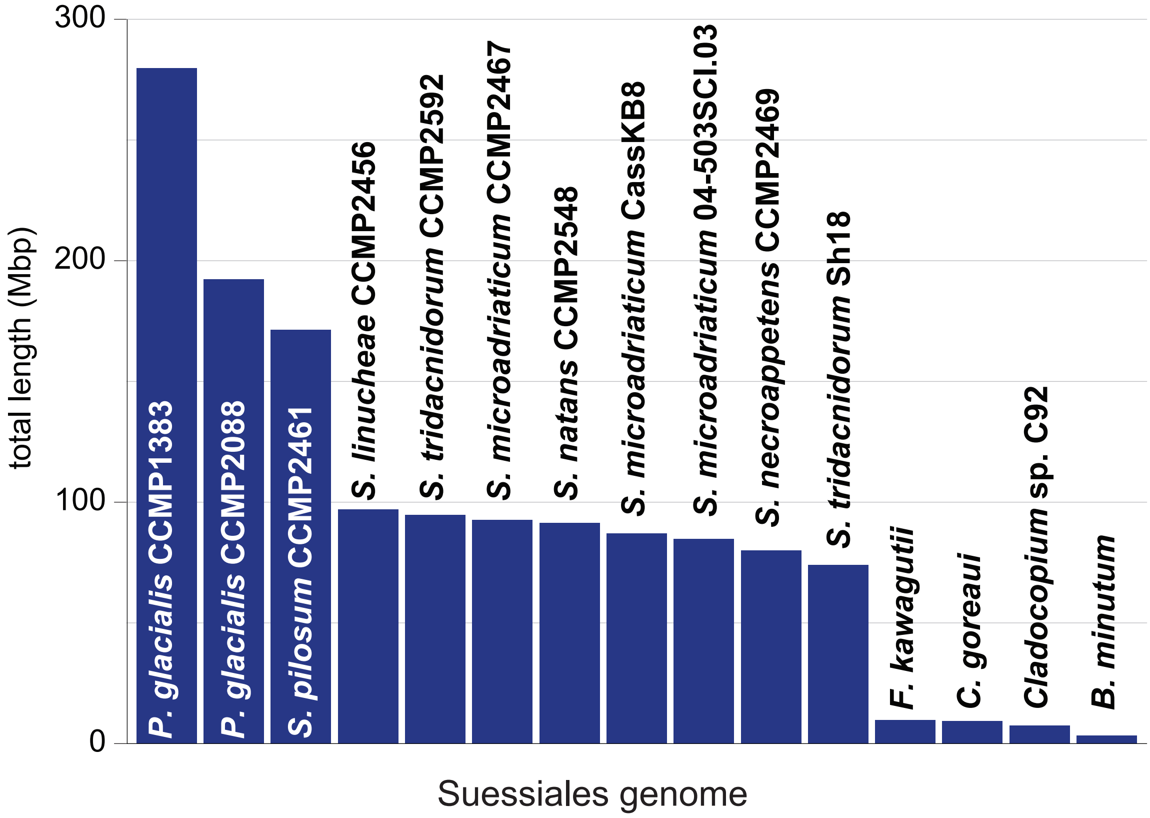

Supplementary Fig. 4. Total length of each analysed Suessiales genome that comprises LINEs, sorted in decreasing order.

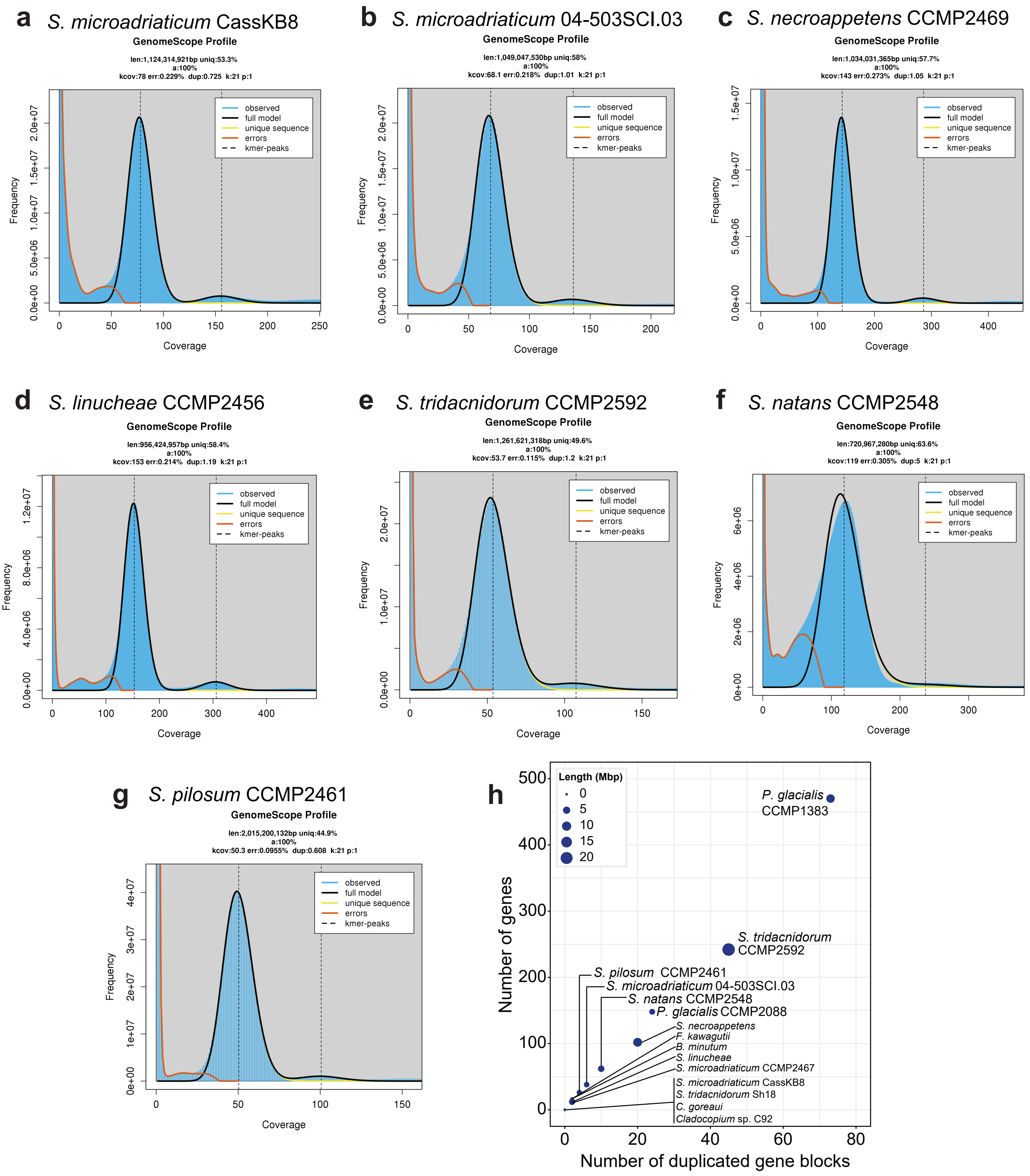

Supplementary Fig. 5. Assessment of *Symbiodinium* genome data generated from this study. (a-g) GenomeScope2 profiles (p:1; haploid) based on 21-mers for each of the seven genome datasets. The single peak of the *k*-mer frequency distribution suggests that the genome is likely haploid (1*n*). (h) Number of the duplicated gene blocks found within each analysed genome (*x*-axis) against the number of implicated genes in those blocks (*y*-axis). The size of the dot is proportional to the added sequence length comprising the duplicated gene blocks, as shown in the top-left legend.

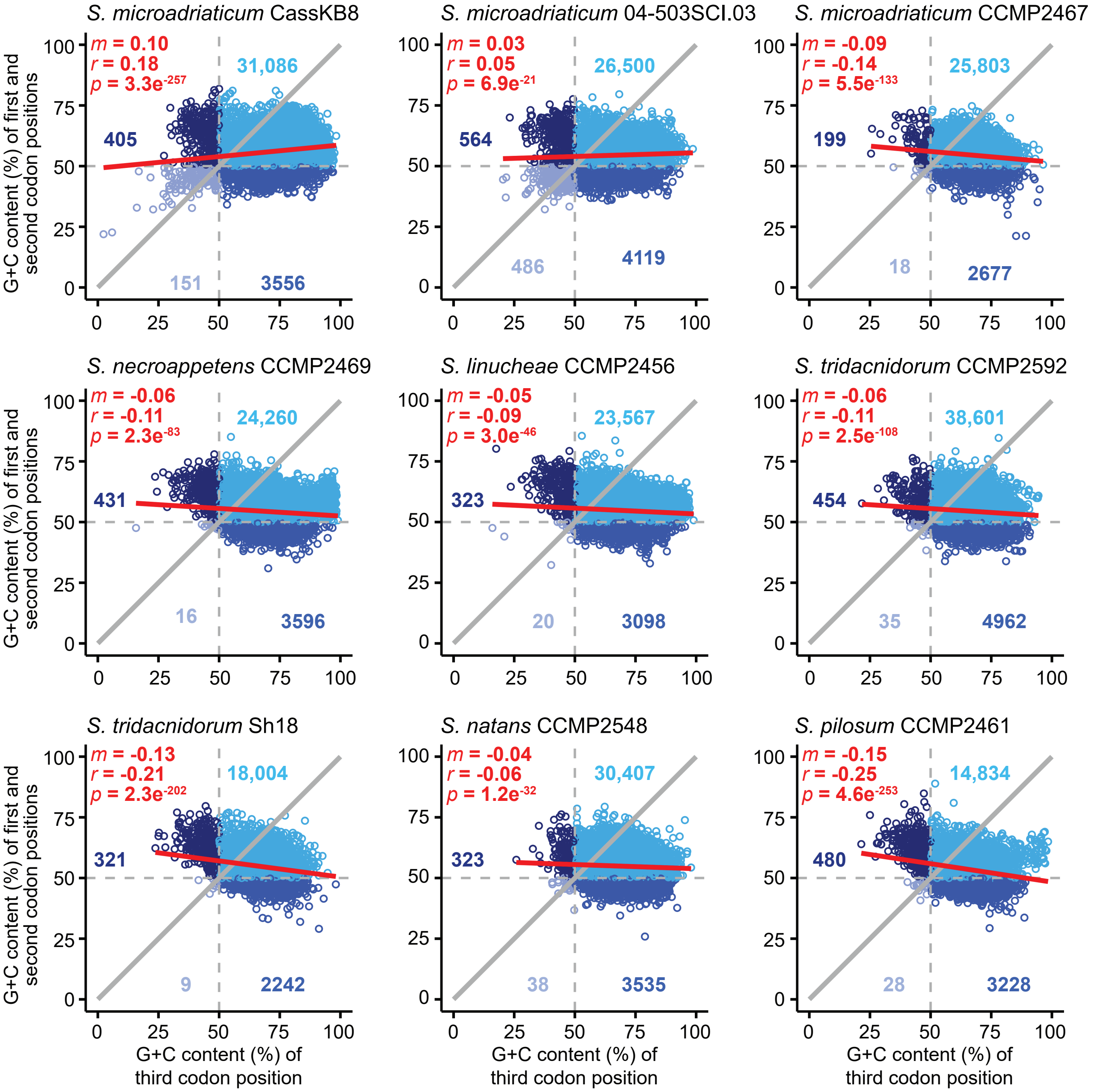

Supplementary Fig. 6. G+C content of third codon position (*x*-axis) against average G+C content of first and second positions (*y*-axis) of full-length CDS in genomes of *Symbiodinium* taxa. The grey diagonal line indicates the values where the nucleotide composition is the same in both metrics, indicative of neutral evolution. The red line represents the regression line estimated from the CDS data; the corresponding slope (*m*), Pearson's correlation coefficient (*r*) and statistical significance (*p*) are shown in red. To highlight overall patterns of nucleotide composition, the plot is split into four quadrants with coordinates at 50% from both axes. Dots falling into each quadrant are coloured in a different shade of blue; the corresponding number of CDS for each quadrant is shown.

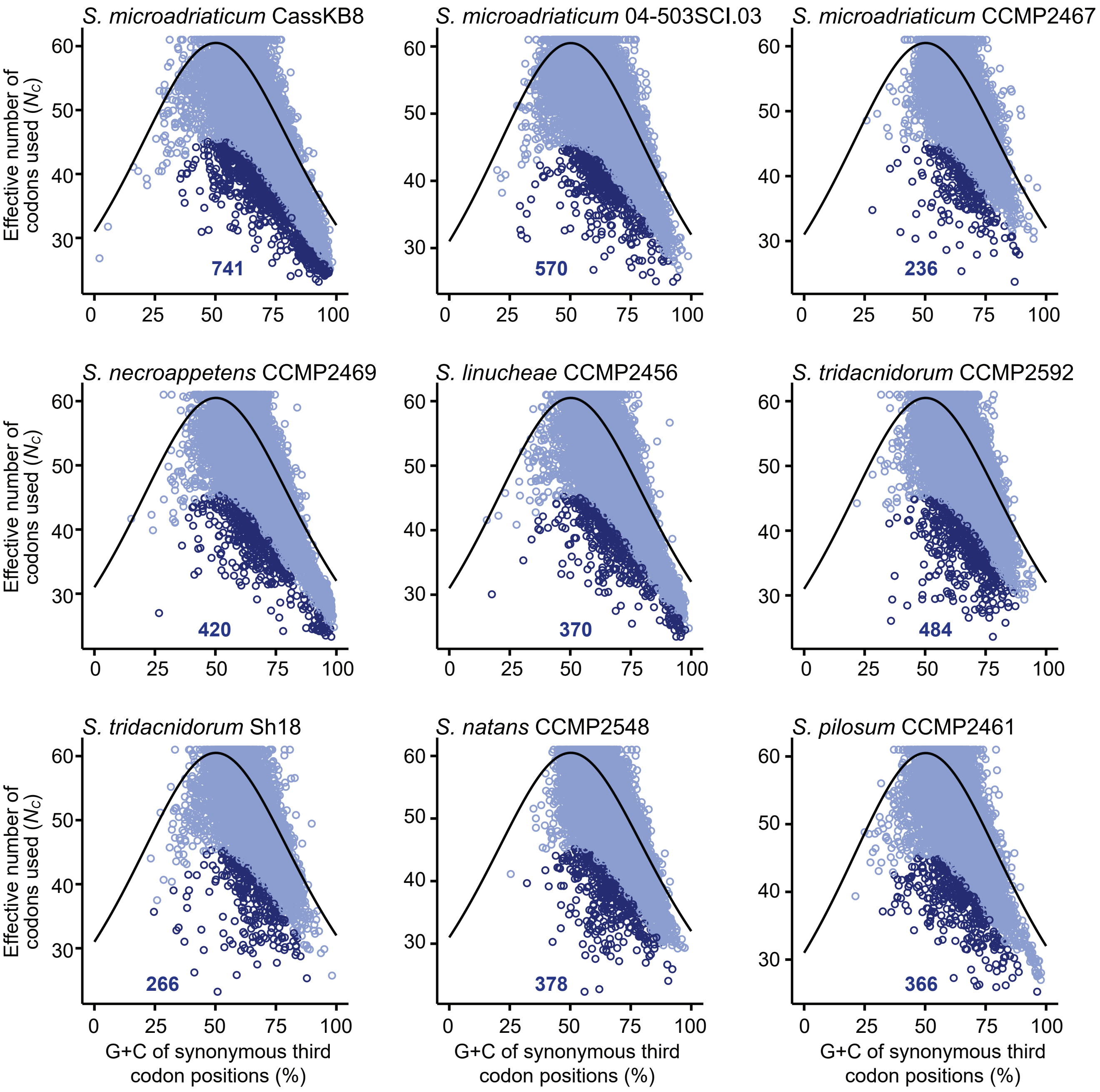

Supplementary Fig. 7. Effective number of codons used (N_C, *y*-axis) as a function of the G+C content of synonymous third codon positions (*x*-axis) in genomes of *Symbiodinium* taxa. The curve line represents the neutral expectation of N_C. CDS with a N_C 25% smaller than the expected are considered to display strong codon usage preference and are highlighted in a darker shade of blue; the corresponding CDS count is shown in each graph.

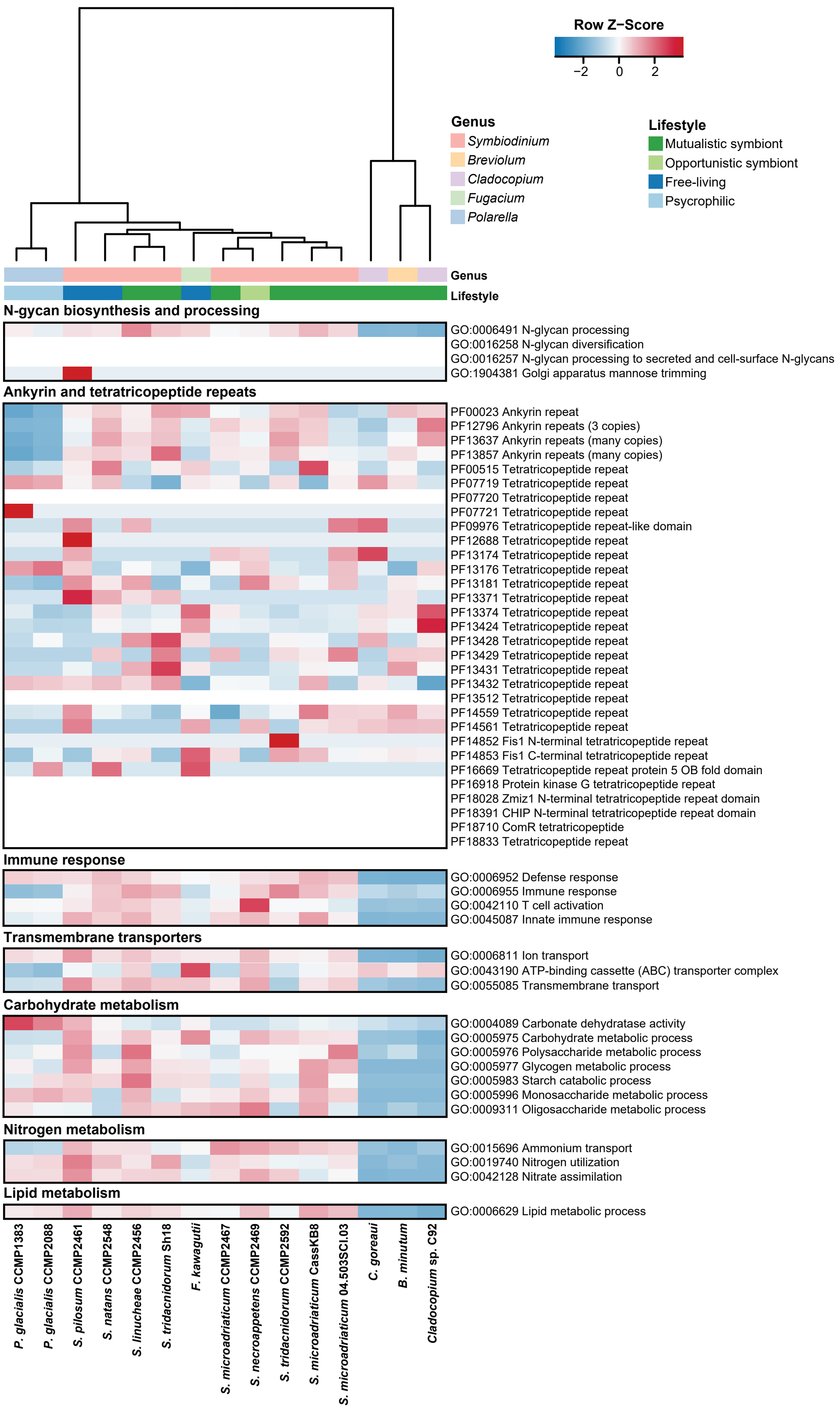

Supplementary Fig. 8. Symbiosis-related gene functions that are encoded in 15 Suessiales genomes. The relative abundance of GO terms and Pfam domains are shown as Z-scores; those with a row of $Z = 0$ are absent in all genomes. Genus and lifestyle are colour-coded based on the legend on the top-right corner.

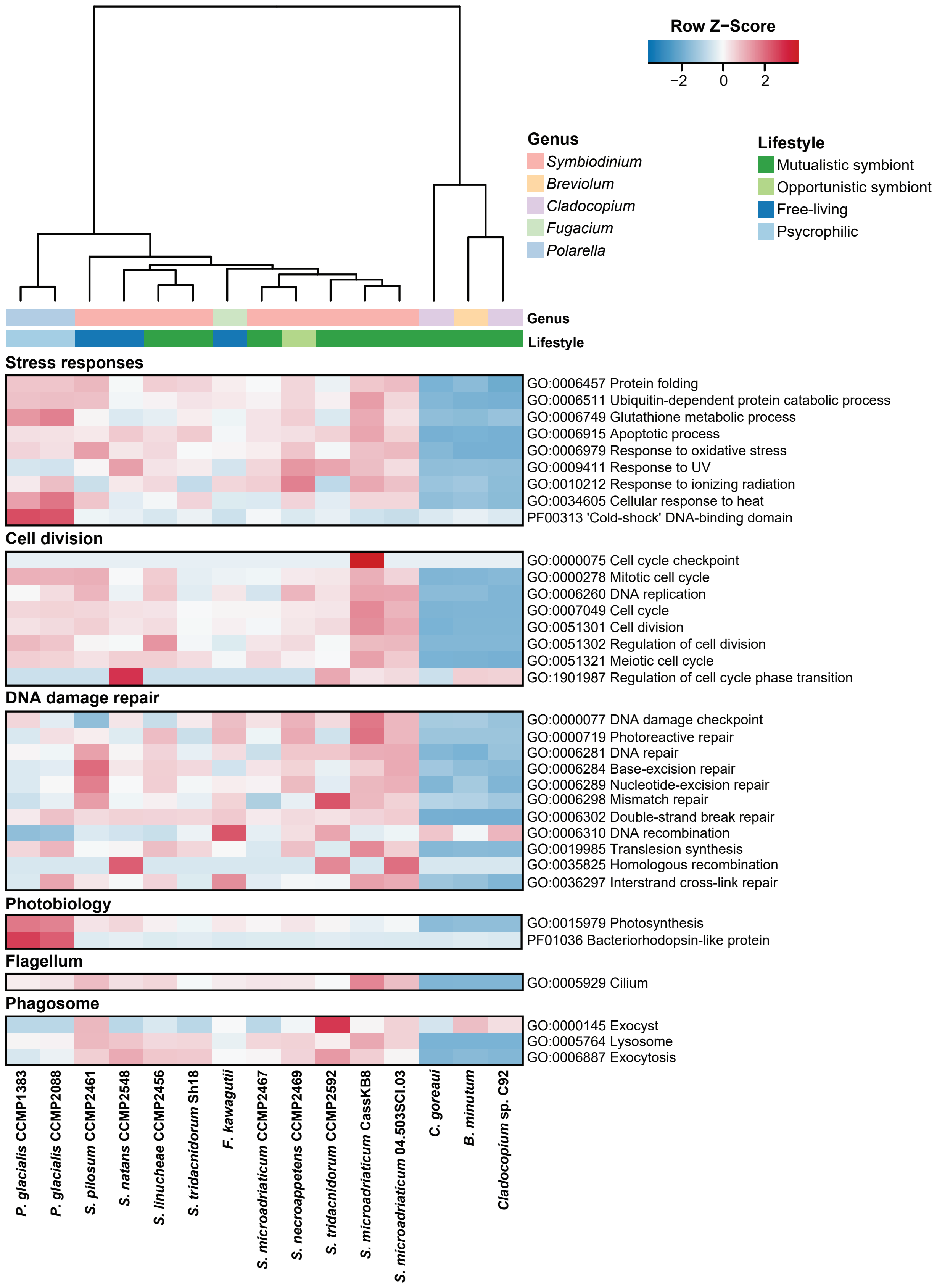

Supplementary Fig. 9. Gene functions related to stress response, cell division, DNA damage repair, photobiology and flagellum, which are encoded in 15 Suessiales genomes. The relative abundance of GO terms and Pfam domains are shown as *Z*-scores. Genus and lifestyle are colour-coded based on the legend on the top-right corner.

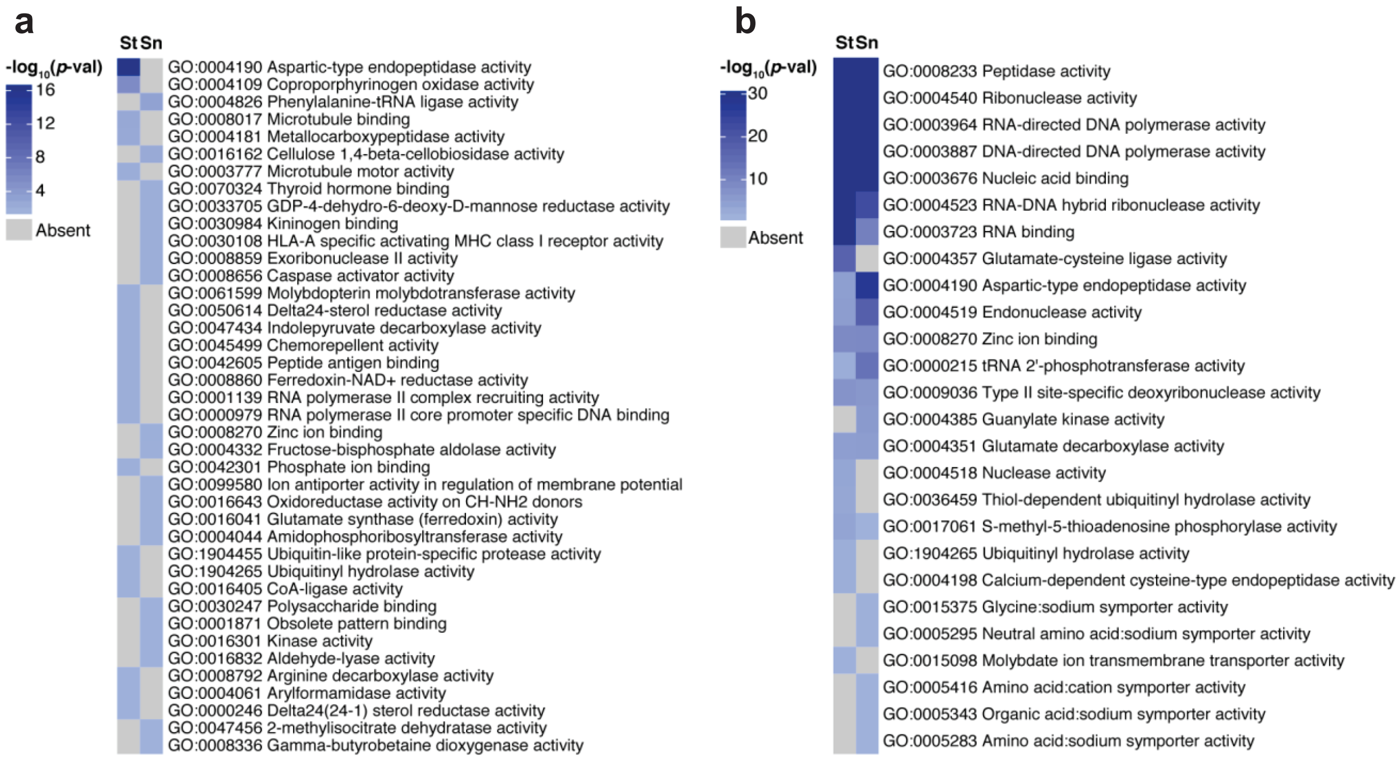

Supplementary Fig. 10. Overrepresented gene functions in *S. tridacnidorum* (St) and in *S. natans* (Sn). GO Molecular Function terms that are enriched in (a) genes with conserved DinoSL relicts in the upstream regions and (b) genes encoding reverse-transcriptase domains.

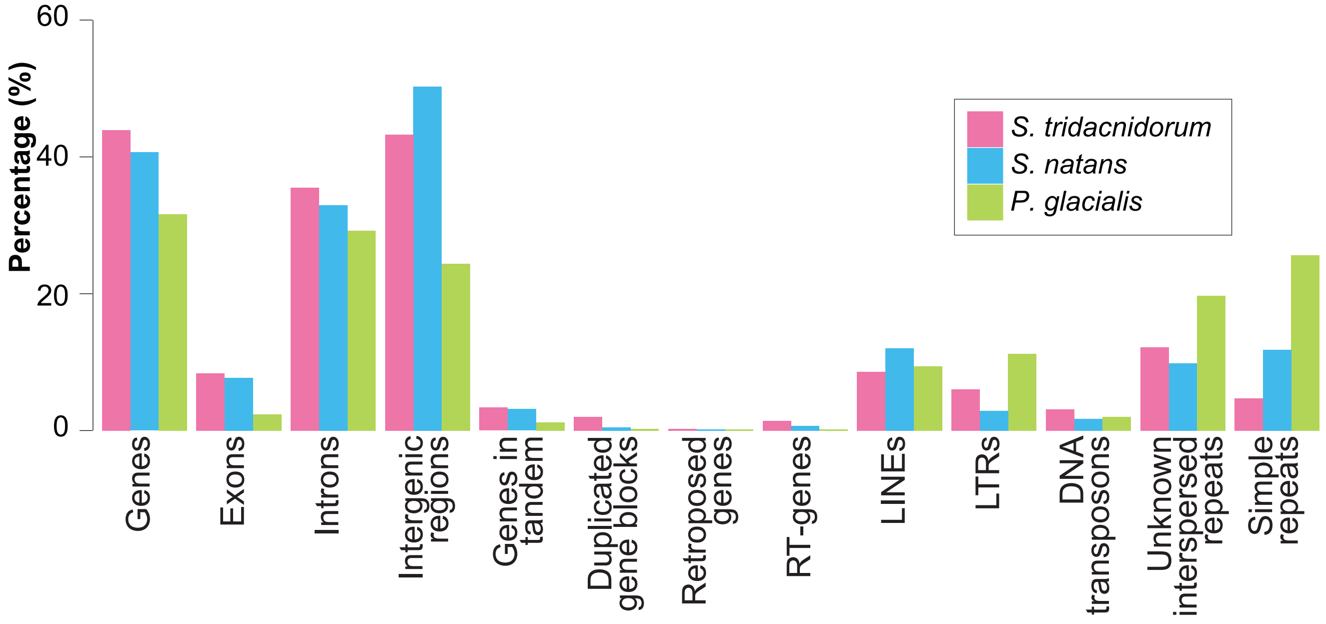

Supplementary Fig. 11. Genome proportion (in percentage of the sequence length) of distinct elements in genomes of *S. tridacnidorum*, *S. natans* and *P. glacialis*.

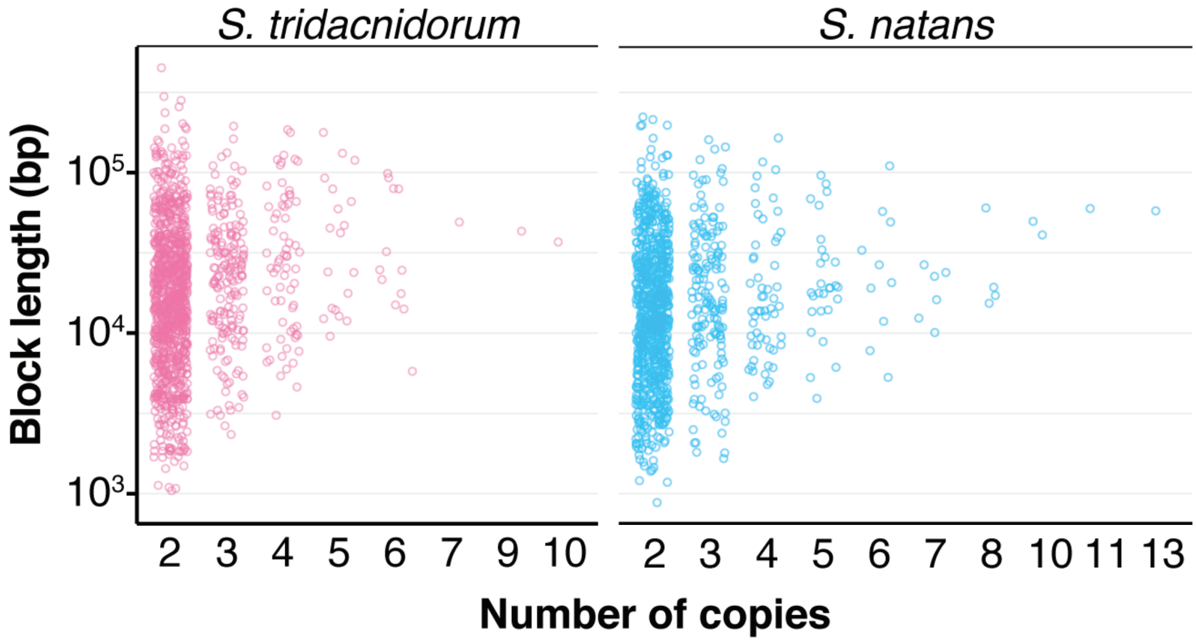

Supplementary Fig. 12. Comparison between genome sequences of *S. tridacnidorum versus S. natans*, showing the distribution of lengths of tandemly repeated gene blocks as a function of the number of gene copies implicated in the blocks.

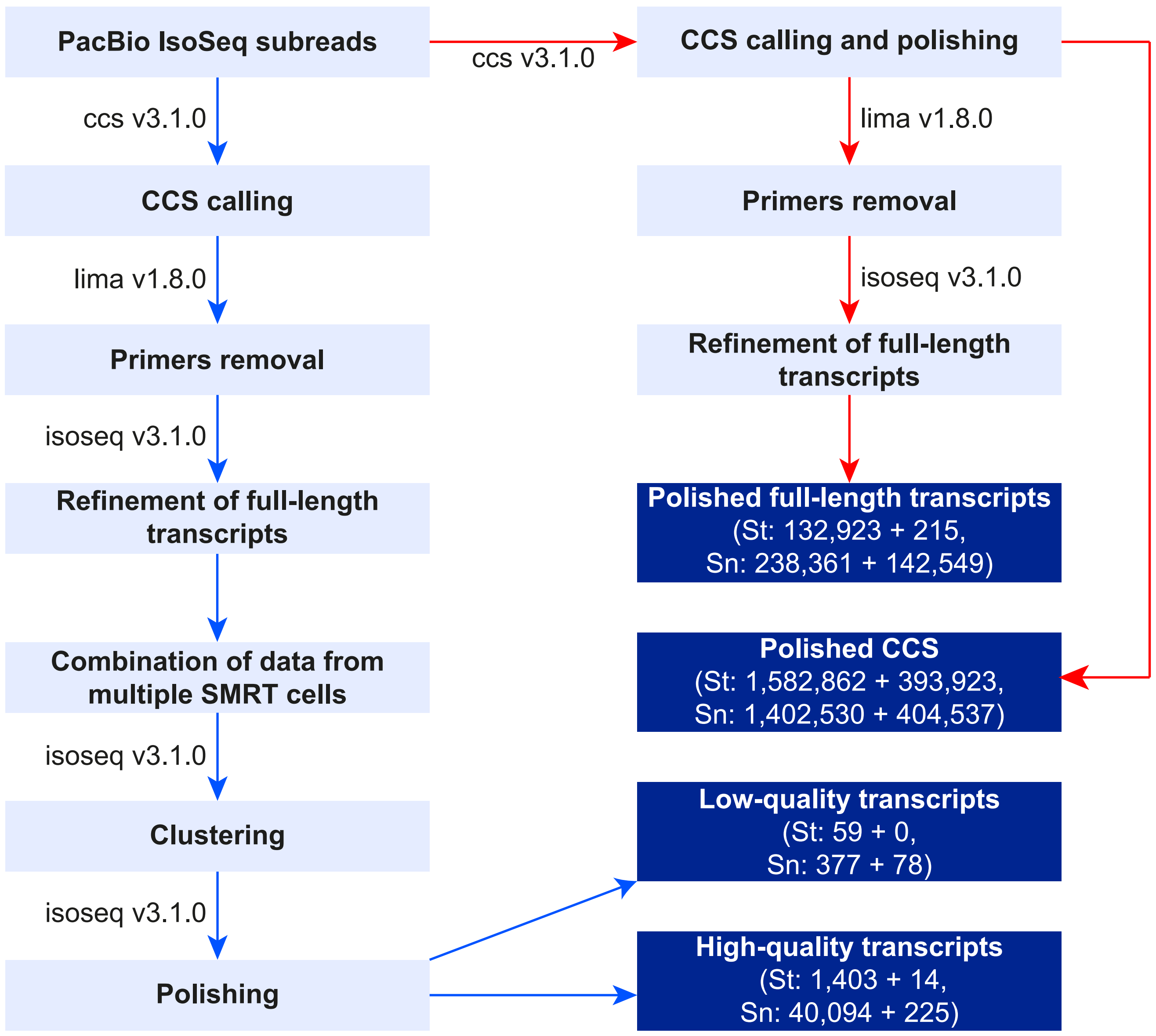

Supplementary Fig. 13. Analytic workflow of Iso-Seq sequencing data and assessment of *k*-mers in the generated genome data. Diagram showing the detailed steps followed to process PacBio IsoSeq data to generate full-length transcript evidence for gene prediction. The traditional IsoSeq 3.1 workflow (blue arrows) was followed to obtain low- and high- quality transcripts. In an alternative approach (red arrows), circular consensus sequences (CCS) were called and polished simultaneously. These polished CCS were further trimmed and refined into full-length transcripts skipping the clustering step. IsoSeq sequences from the DinoSL library were processed apart from the other libraries. Boxes in dark blue represent the transcript evidence subsequently used for gene prediction and the values in parentheses show the corresponding number of sequences from the standard (left) + the DinoSL (right) libraries for both *S. tridacnidorum* (St) and *S. natans* (Sn).

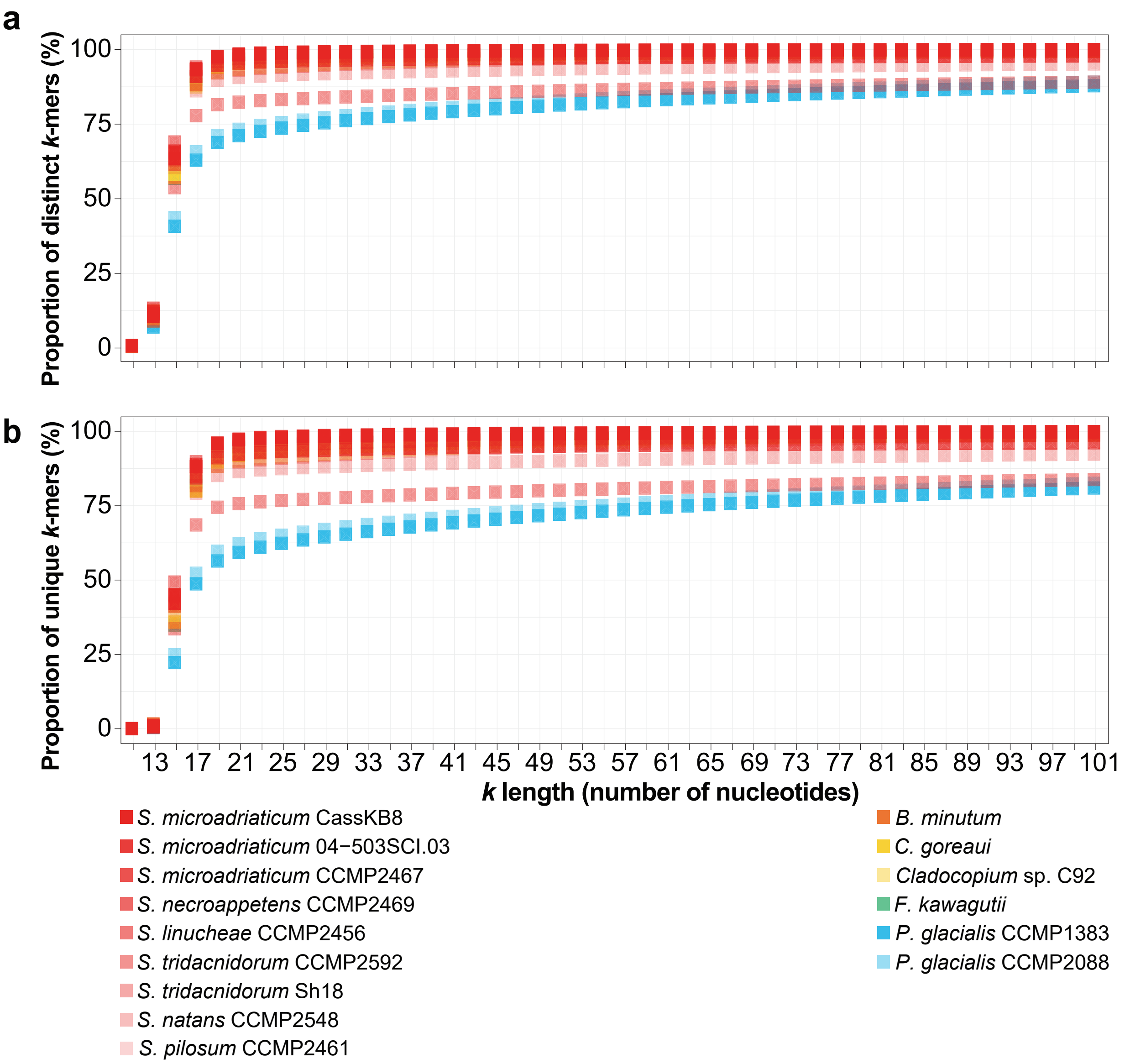

Supplementary Fig. 14. Proportion of (a) distinct and (b) unique *k*-mers observed in the genome data for each isolate across different values of *k* length.
